# Hypervalent Iodine Reagents in Palladium-Catalyzed Oxidative Cross-Coupling Reactions

**DOI:** 10.3389/fchem.2020.00705

**Published:** 2020-09-29

**Authors:** Samata E. Shetgaonkar, Fateh V. Singh

**Affiliations:** Chemistry Division, School of Advanced Science, Vellore Institute of Technology, Chennai, India

**Keywords:** hypervalent iodine reagents, palladium, oxidant, catalyst, bond formation

## Abstract

Hypervalent iodine compounds are valuable and versatile reagents in synthetic organic chemistry, generating a diverse array of useful organic molecules. Owing to their non-toxic and environmentally friendly features, these reagents find potential applications in various oxidative functionalization reactions. In recent years, the use of hypervalent iodine reagents in palladium-catalyzed transformations has been widely studied as they are strong electrophiles and powerful oxidizing agents. For instance, extensive work has been carried out in the field of C–H bond functionalization via Pd-catalysis using hypervalent iodine reagents as oxidants. In addition, nowadays, iodine(III) reagents have been frequently employed as arylating agents in Pd-catalyzed C–H arylation or Heck-type cross-coupling reactions. In this review, recent advancements in the area of palladium-catalyzed oxidative cross-coupling reactions using hypervalent iodine reagents are summarized in detail.

**Graphical Abstract:**
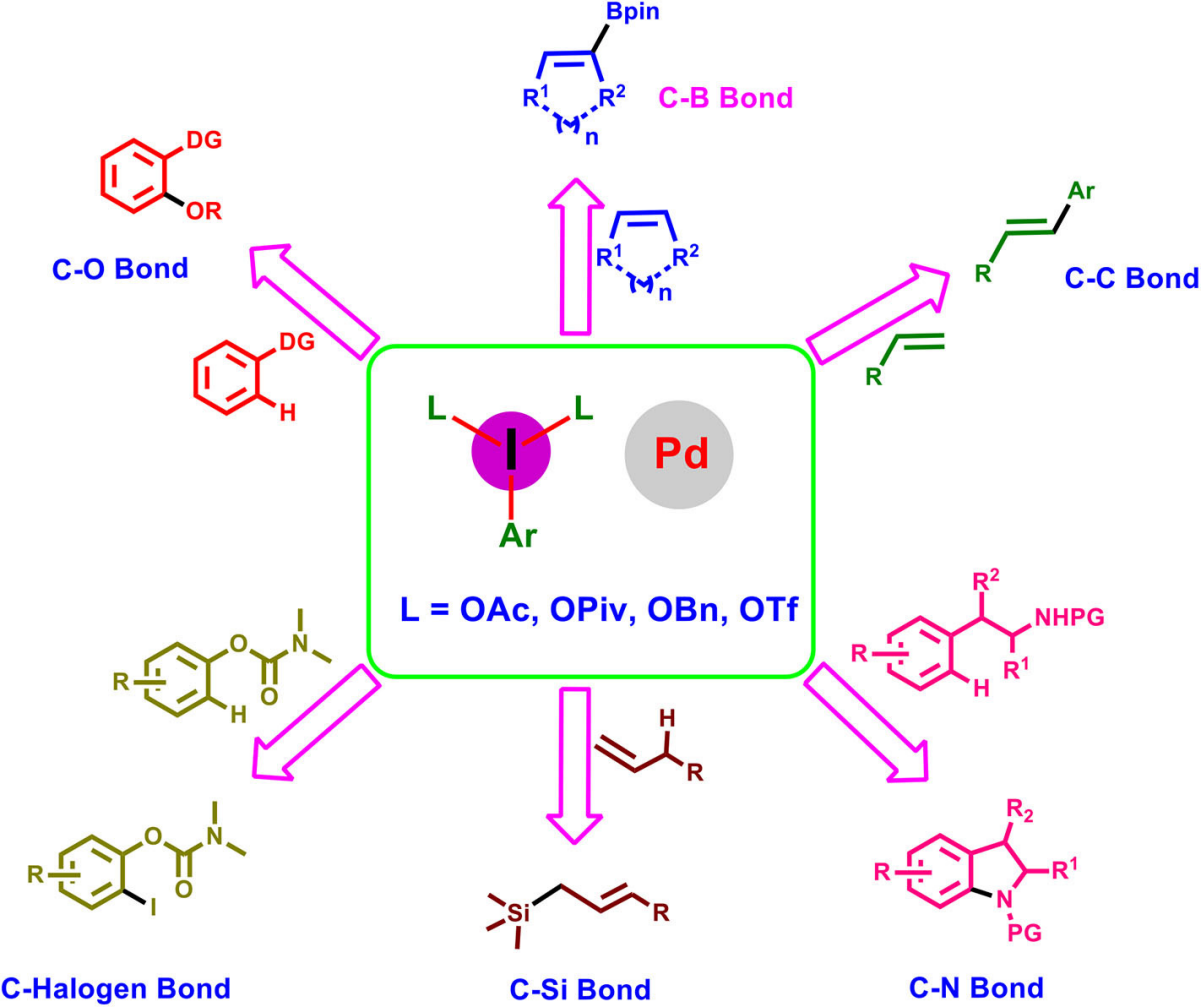
Representative palladium-catalyzed reactions using hypervalent iodine reagents.

## Introduction

Hypervalent iodine compounds are ubiquitous in organic synthesis since they have emerged as efficient and environmentally benign reagents for academic and industrial research (Zhdankin, 2014; Yoshimura and Zhdankin, 2016; Singh and Wirth, 2018). They are non-toxic, easily prepared, stable and alternative to metal-derived oxidants/catalysts in various oxidative transformations (Yusubov and Zhdankin, 2012; Singh and Wirth, 2014a,b, 2017; Mangaonkar et al., 2018; Singh et al., 2018a; Mangaonkar and Singh, 2019). Several research papers, book chapters and review articles have been published covering various aspects of hypervalent iodine compounds as reagents or as catalysts in α-functionalization of carbonyl compounds (Merritt and Olofsson, 2011; Dong et al., 2014), cyclizations (Singh and Wirth, 2011, 2012; Singh and Mangaonkar, 2018; Singh et al., 2018b), oxidative rearrangements (Singh et al., 2012; Singh and Wirth, 2013; Maertens and Canesi, 2015), alkene difunctionalizations (Li et al., 2018; Lee et al., 2019) and atom-transfer reactions (Li Y. et al., 2016). The inherent ability of hypervalent iodine reagents to act as oxidants as well as ligand transfer reagents is the key to the significant progress achieved in this area.

**Figure 1:**
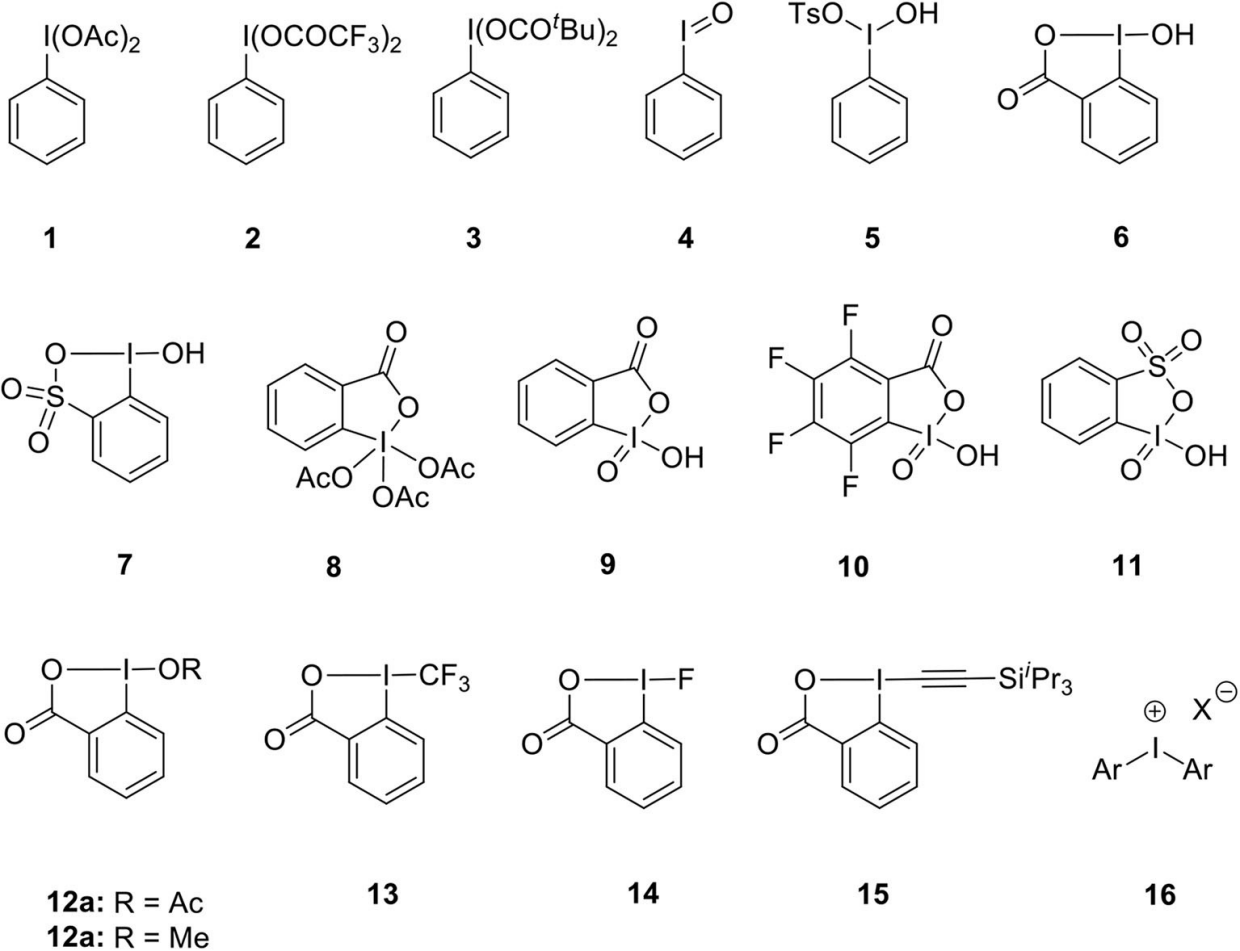
Examples of hypervalent iodine(III)/(V) reagents **1–16**.

Representative examples of various hypervalent iodine(III)/(V) reagents commonly used as oxidants or atom transfer reagents are listed in Figure 1. Iodobenzenediacetate (PIDA) **1**, [bis(trifluoroacetoxy)iodo] benzenes (PIFA) **2** and iodosobenzene dipivalate **3** are routinely used iodine(III) oxidants (Wirth, 2005; Zhdankin, 2009). Apart from this, other oxidizing agents such as iodosobenzene **4**, Koser reagent **5**, 2-iodosobenzoic acid (IBA) **6** and l-hydroxy-1*H*-1,2,3-benziodoxathiole 3,3-dioxide **7** were used in various oxidative transformations (Singh and Wirth, 2014a). Dess–Martin periodinane (DMP) **8** and 2-iodoxybenzoic acid (IBX) **9** are extensively used cyclic iodine(V) reagents (Tohma and Kita, 2004; Uyanik and Ishihara, 2009). 2,3,4,5-tetrafluoro-6-iodoxybenzoic acid (FIBX) **10** having higher reactivity than IBX **9** was synthesized by Richardson et al. (2007). Zhdankin's group prepared 2-iodoxybenzenesulfonic acid (IBS) **11** via oxone-mediated oxidation of 2-iodobenzenesulfonic acid (Koposov et al., 2006). Another important aspect of iodine(III)/(V) reagents that has received considerable attention is to act as electrophilic synthons for functional-group transfer reactions. For example, benziodoxol(on)e reagents **12**–**15** have been employed as a source of nucleophilic groups such as acetate, trifluoromethyl, fluorides, and alkynes in various transformations (Li Y. et al., 2016). Diaryliodonium salts **16** being electrophilic and a naturally good leaving group, are widely used as arylating reagents (Merritt and Olofsson, 2009).

However, these reagents do have limitations such as stoichiometric generation of aryliodides as a byproduct, limited solubility in common organic solvents, and requirement of an excess amount of reagents thus lowering atom economy dramatically. For instance, IBA **6** is less explored due to its lower reactivity while DMP **8** is expensive and moisture sensitive. IBX **9** is associated with drawbacks such as an explosive nature, poor solubility in common organic solvents, and requirement of high reaction temperature which limits its synthetic applications to some extent (Singh and Wirth, 2014b). Also, IBS **11** is thermally unstable and highly reactive toward organic solvents, like acetonitrile, dimethyl sulfoxide, and methanol and readily decomposed into stable thia-analog of IBA **7** (Koposov et al., 2006). Although there are some drawbacks associated with these reagents, there is still great scope for these compounds as potential alternate candidates for toxic heavy-metal oxidants such as mercury, thallium, and lead based reagents (Silva L. F, 2002; Wirth et al., 2002).

On the other hand, palladium as a catalyst has become a fundamental part of various coupling reactions such as Suzuki-Miyaura, Heck, Buchwald–Hartwig, Sonagashira, and Negishi generating diverse array of useful molecules (Biffis et al., 2018). Hypervalent iodine reagents in palladium-catalyzed reactions constitute an interesting area of research in organic synthesis. Deprez and Sanford published the first review article explaining the unique reactivity of hypervalent iodine reagents in Pd-catalyzed reactions in 2007 (Deprez and Sanford, 2007). Later, the 2015 section of the review that describes the key role of polyvalent iodine reagents in high valent palladium chemistry was published by Wengryniuk's group (Silva et al., 2017). Owing to their excellent oxidizing and electrophilic nature, hypervalent iodine reagents reacts with palladium complexes and promotes reactions via Pd(0/II) or Pd(II/IV) redox cycles. For instance, iodine(III) reagents have been employed as a substitute for aryl halides in various Pd(0)/(II)-catalyzed cross-coupling reactions (Deprez and Sanford, 2007). Also, various Pd-catalyzed ligand transfer reactions employ few of these reagents as a source of aryl, alkynyl, and heteroatom ligands. Although substantial work has been carried out in Pd(0)/(II)-catalysis in the past, catalytic reactions via Pd(II)/(IV) pathway were less explored. However, with the aid of hypervalent iodine oxidants, Pd(II)/(IV)-catalysis have seen tremendous development in the last couple of decades enabling various synthetic transformations earlier inaccessible via traditional catalytic manifolds. For example, C–H functionalizations have become a powerful tool to construct new C–C and C–heteroatom bonds using Pd(II)/(IV)-catalysis.

Within this context, the present review focuses on the recent advancement accomplished in palladium-catalyzed transformations using hypervalent iodine reagents, highlighting its potential synthetic applications and mechanistic aspects. The article is classified on the basis of bonds formed such as C–O, C–N, C–C, C–Si, C–B, and C–halogen bonds and finally alkene difunctionalization reactions.

## C–O Bond Formation

Palladium-catalyzed ligand assisted C–H functionalization has been proven to be one of the best, most atom economical, and effective tools in organic synthesis for the introduction of variable functional groups at the unactivated arene/alkane C–H bonds. Extensive research has been carried out by several groups in the field of C–O bond formation via palladium catalysis using hypervalent iodine reagents as oxidant or heteroatom ligand. Among them, Pd-catalyzed C–H oxidative cyclization, C–H acyloxylation, C–H alkoxylation and allylic oxidation constitutes important pathways in C–O bond formation reactions directed by directing functional groups such as oxime ether, oxazoline, amide, pyridine and pyrimidine, etc.

### C–H Cyclization

Significant progress has been made in the field of Pd-catalyzed oxidative cyclization reactions using hypervalent iodine reagents, giving access to several oxygen-containing heterocycles. In 2010, Wang et al. developed a novel method for the construction of dihydrobenzofurans via Pd(OAc)_2_-catalyzed hydroxyl group-directed C–H activation/C–O cyclization reaction in the presence of (diacetoxyiodo)benzene **1** as a terminal oxidant and promoted by base Li_2_CO_3_. Moreover, the scope of the reaction was extended to the preparation of important scaffolds such as spirocyclic dihydrobenzofurans (Wang X. et al., 2010).

**Scheme 1:**
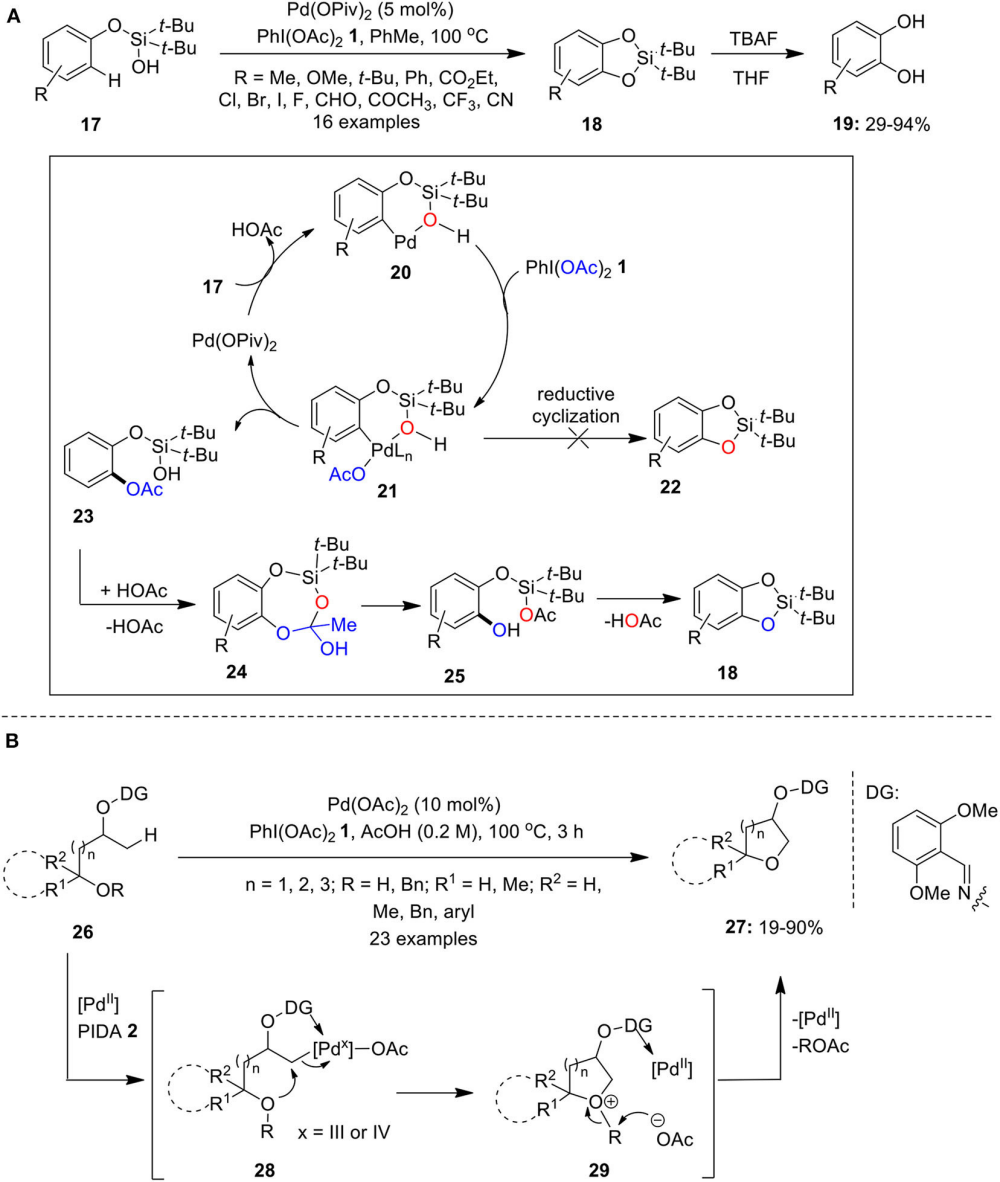
**(A)** Pd(II)-catalyzed synthesis of substituted catechols **19** using PhI(OAc)_2_ **1** as an oxidant and **(B)** Pd(II)-catalyzed synthesis of cyclic ethers **27** using PhI(OAc)_2_ **1** as an oxidant.

Later, Gevorgyan's group demonstrated intramolecular silanol group-directed C–H oxygenation of phenoxy silanols **17** employing Pd(OPiv)_2_ as catalyst and PhI(OAc)_2_ **1** as an oxidant (Huang et al., 2011). This reaction involves the initial formation of cyclic silicon-protected catechols **18** which upon desilylation with TBAF/THF provides substituted catechols **19** (Scheme 1A). The plausible catalytic cycle involves initial coordination of Pd with silanols **17** to form palladacycle **20** accompanied by PIDA-mediated oxidation to forge Pd(IV)-intermediate **21**. Next, intermediate **21** is transformed into acetoxylated intermediate **23** via reductive acetoxylation and regenerates back palladium catalyst. Finally, acid-catalyzed transesterification of **24** gives **25** which subsequently loose acetic acid to form cyclic silyl protected catechols **18** followed by subsequent desilylation with TBAF furnishes catechols **19**. Further formation of products **22** through direct C–O reductive cyclization was eliminated based on ^18^O-labeling studies. Further reactions featured excellent site selectivity and broad substrates scope, particularly, electron-rich substrates reacted much faster and provided high yields. Another interesting work published by Gevorgyan's research group employing C–H oxygenation strategy is the convenient synthesis of oxasilacycles from substituted benzyl-silanols using combination of Pd(OAc)_2_ and PhI(OAc)_2_ **1** in PhCF_3_ at 100°C (Huang et al., 2012).

Furthermore, Thompson et al. reported the preparation of cyclic ethers **27** via Pd-catalyzed oxime masked-alcohol directed dehydrogenative annulation of sp^3^ C–H bonds of substrates **26** using PhI(OAc)_2_ **1** as an oxidant (Thompson et al., 2015). The reaction occurs selectively at the β-position and substrates **26** having primary, secondary, and tertiary hydroxyl group worked smoothly under the standard conditions (Scheme 1B). The reaction could proceed via C–H palladation, followed by oxidation of Pd to higher oxidation state species **28** and a subsequent intramolecular S_N_2 reaction to form oxonium intermediate **29**. Finally, cyclic ethers **27** were obtained through deprotonation or debenzylation and regenerate Pd-catalyst.

**Scheme 2:**
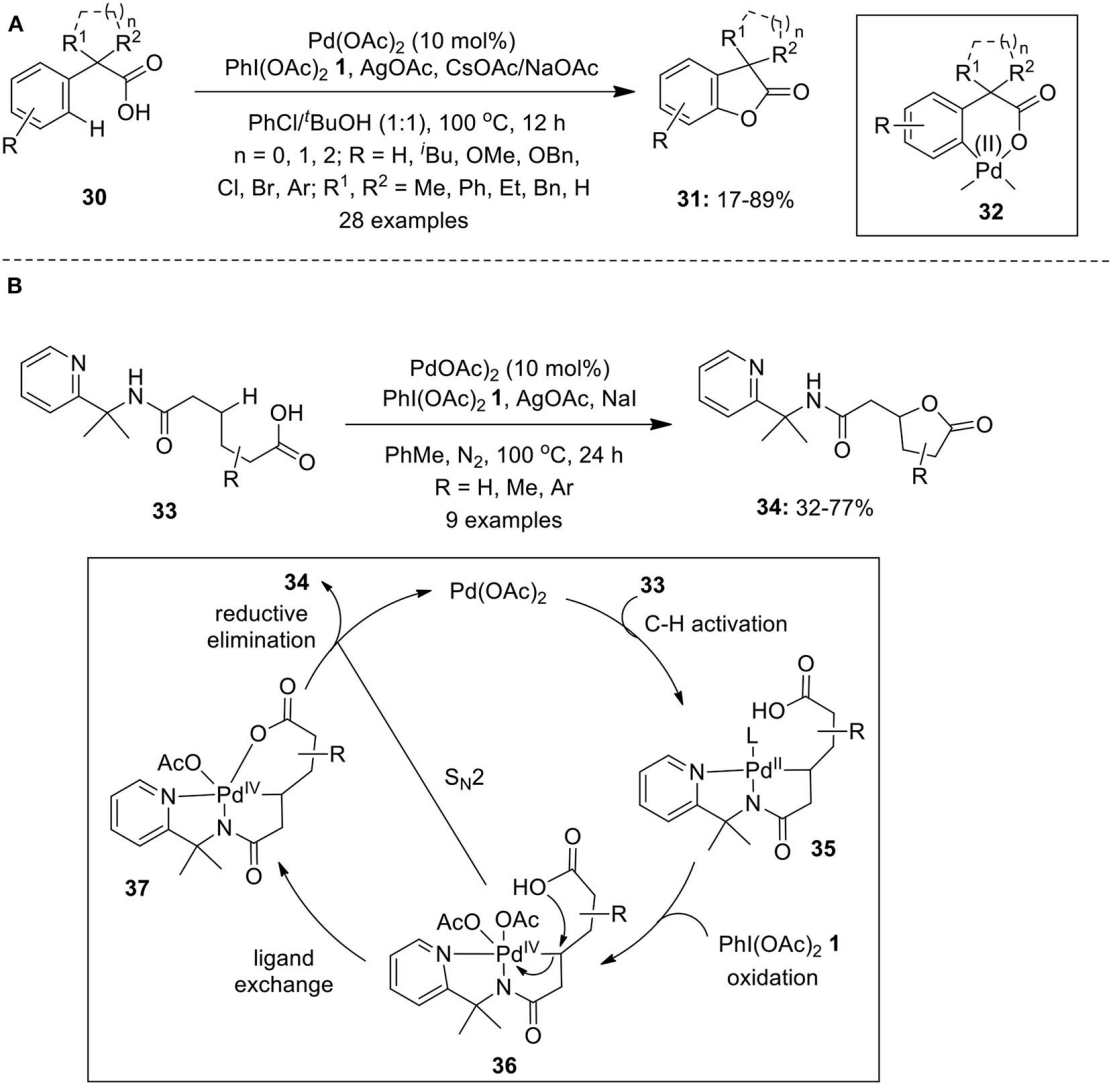
**(A)** Pd(II)-catalyzed α,α-disubstituted benzofuran-2-ones **31** using PhI(OAc)_2_ **1** and **(B)** Pd(II)-catalyzed synthesis of γ-lactones **34** using oxidant PhI(OAc)_2_ **1**.

Yang et al. reported Pd-catalyzed intramolecular lactonization of α, α-disubstituted arylacetic acids **30** in the presence of PhI(OAc)_2_ **1** to furnish a series of α, α-disubstituted benzofuran-2-ones **31** in variable yields (Yang et al., 2013). The catalytic system composed of Pd(OAc)_2_ and the combination of NaOAc and CsOAc along with AgOAc as most effective bases (Scheme 2A). The authors proposed that C–H cleavage occurs via concerted metalation deprotonation to form six-membered palladacycle **32** which further undergoes oxidation and subsequent reductive elimination to afford **31**. Similar catalytic C–H activation/C–O formation methods to construct functionalized benzofuranones (Cheng et al., 2013) and biaryl lactones (Li et al., 2013) were also developed employing acetyl-protected glycine (Ac-Gly-OH) as requisite ligand along with base KOAc in *t*-BuOH.

In 2016, Shi's group described a concise route to access γ-lactones **34** featuring Pd(II)-catalyzed PIP auxiliary-directed lactonization of unactivated methylene C(sp^3^)–H bonds using oxidant PIDA **1** (Liu and Shi, 2016). The lactonization of aliphatic acids **33** bearing various substituents on the alkyl chain proceeded remarkably well to furnish anticipated γ-lactones **34** in 32–77% yields (Scheme 2B). The probable mechanism for the lactonization of aliphatic acids **32** involves formation of five-membered palladacycle **35** through Pd-catalyzed C–H activation assisted by bidentate auxiliary. In the presence of PhI(OAc)_2_ **1**, palladacycle **35** underwent oxidation to give Pd(IV) intermediate **36** followed by ligand exchange to form **37** which finally undergoes reductive elimination to release target product **34** along with Pd(II) catalyst to complete the catalytic cycle (**Scheme 11**). Another path to generate lactone **34** is via direct S_N_2-type attack by carboxylate group onto the Pd(IV)–C bond of **36** (Scheme 2B).

### C(sp^2^/sp^3^)–H Acyloxylation

Over the years, C–H acyloxylation has made tremendous progress in transforming various unactivated sp^2^ and sp^3^ hybridized C–H bonds into valuable C–O bonds using palladium catalysts and iodine(III) reagents as oxidants. This method provides a concise route for the introduction of valuable ester functionality on to the aromatic or aliphatic substrates under mild conditions. Several C(sp^2^)–H acyloxylation protocols have been developed employing Pd(OAc)_2_/PhI(OAc)_2_ **1** catalytic systems using a variety of directing groups such as pyrimidine (Gu et al., 2009), 8-aminoquinoline (Gou et al., 2009), oxime (Neufeldt and Sanford, 2010; Ren et al., 2015), pyridyldiisopropylsilyl (Chernyak et al., 2010; Gulevich et al., 2012), and 1,2,3-triazoles-pyridine (Ye et al., 2013).

**Scheme 3:**
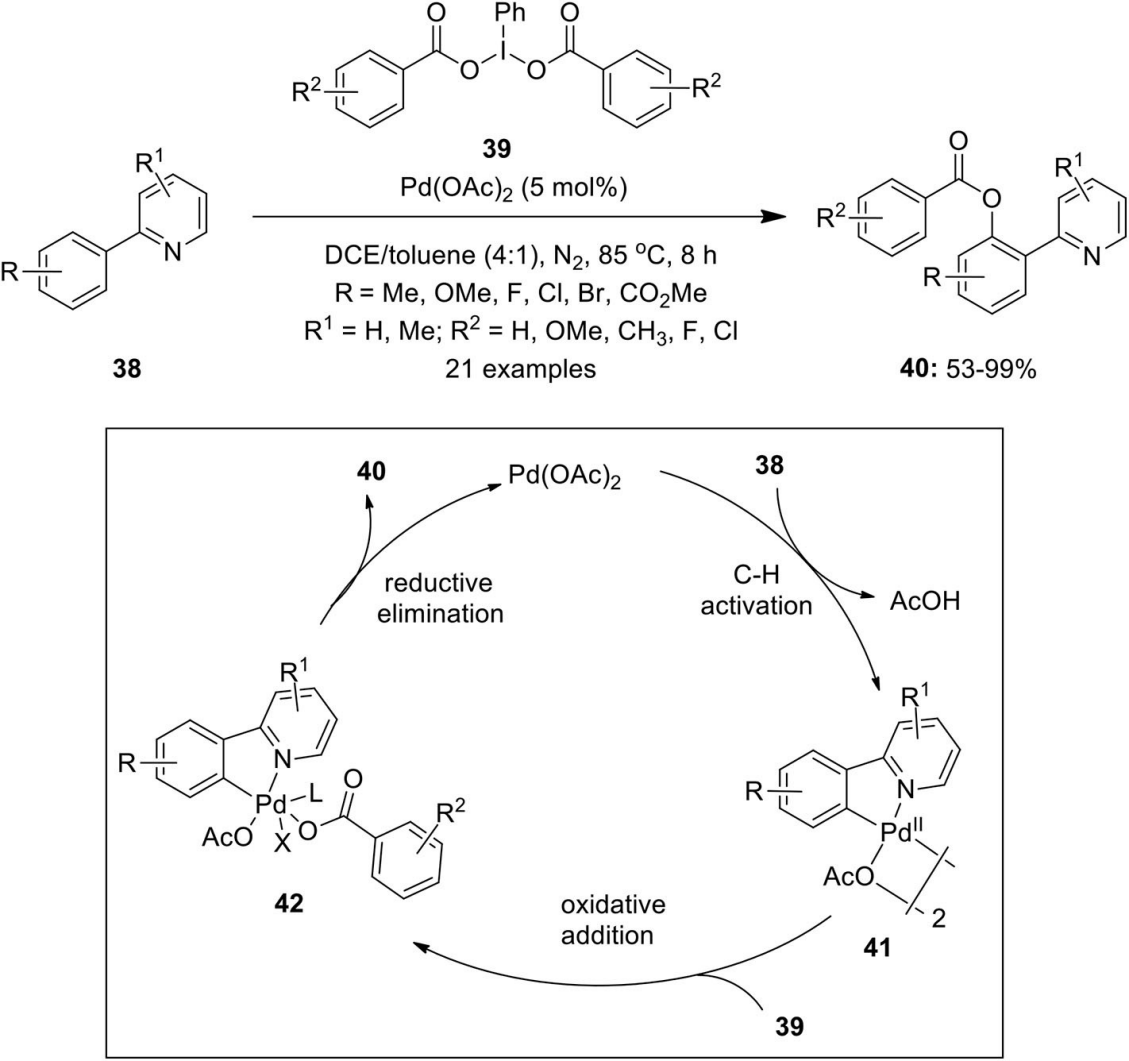
Pd(II)-catalyzed *ortho* C–H benzoxylation of 2-arylpyridine**s 38** using substituted iodobenzene dibenzoates derivatives **39** as oxidant and source of benzoxyl group.

Furthermore, a regioselective protocol featuring Pd(II)-catalyzed C–H benzoxylation of 2-arylpyridines **38** was performed by Zhang et al. affording mono-benzoxylation products **40** in moderate to excellent yields (Zhang et al., 2015). They employed easily accessible iodobenzene dibenzoate derivatives **39** as oxidants and sources of the benzoxyl group (Scheme 3). Furthermore, the present method was successfully applied for the benzoxylation of 2-thienyl pyridines to yield 3-benzoxylated thiophenes in high yields. The plausible mechanism proceeds via pyridyl-assisted C–H activation of substrates **38** with a palladium catalyst to form complex **41** which undergoes further oxidative addition with **39** to form high oxidation state complex **42**. Finally, the reductive elimination of **42** gives desired product **40** and regenerates the palladium catalyst to complete the catalytic cycle.

Although substantial development has been accomplished in the Pd-catalyzed ligand-directed C–H oxygenation reactions, the non-chelate assisted transformations are rarely explored. For instance, Pd-catalyzed C–H acetoxylation of arenes devoid of directing groups remains a challenge as it leads to the formation of mixtures of isomers. In this context, Emmert et al. developed non-chelate assisted palladium-catalyzed C–H acetoxylation of simple arenes using pyridine (Emmert et al., 2011) and acridine (Cook et al., 2013) as ancillary ligands in combination with hypervalent iodine reagents as oxidants.

Furthermore, the Pd-catalyzed allylic C–H acyloxylation represents one of the efficient methods in C–H functionalization reactions. Pilarski et al. have presented an excellent protocol for the preparation of allylic acetates or allylic benzoates via Pd-catalyzed allylic C–H acetoxylation/benzoyloxylation using PhI(OAc)_2_ **1** or PhI(OBz)_2_ as oxidant and source of acyloxy group (Pilarski et al., 2009). Later, the same group described conversion of alkenes into allylic trifluoroacetates via Pd-catalyzed C–H trifluoroacetoxylation employing PhI(OCOCF_3_)_2_ **2** as an oxidant and trifluoroacetoxy source (Alam et al., 2012).

**Scheme 4:**
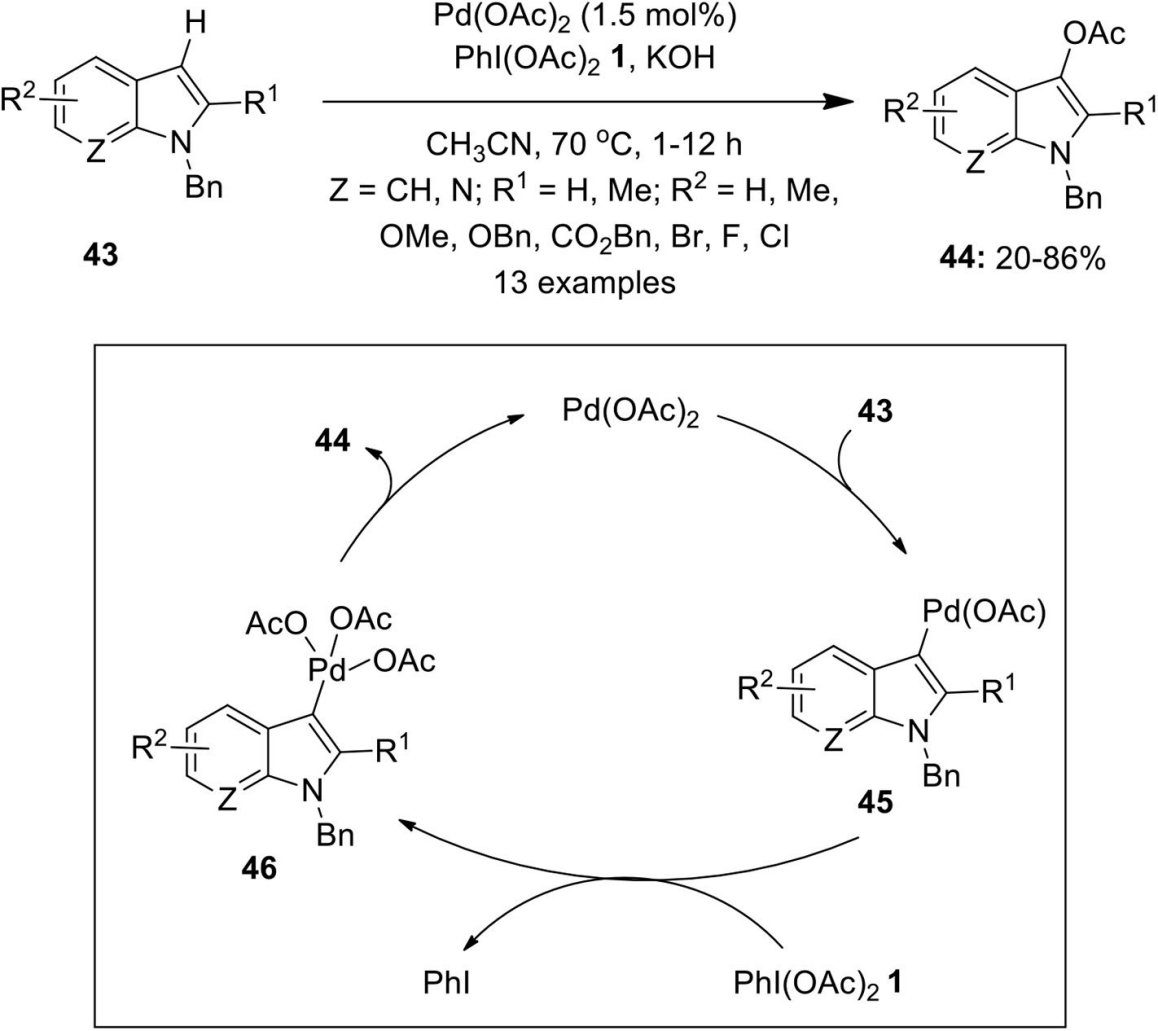
Pd(II)-catalyzed C3-acetoxylation of substituted indoles **43** to afford C3-acetoxylated indoles **44** using PhI(OAc)_2_ **1**.

Regioselective C–H functionalization of indoles has been found to be a straight forward route to access biologically important 3-acetoxyindoles. In this regard, Suna's and Kwong's groups independently reported the synthesis of 3-acetoxyindoles via direct C3-oxidation of indole derivatives catalyzed by Pd(OAc)_2_ (2–5 mol%) in the presence of terminal oxidant PhI(OAc)_2_ **1** (Mutule et al., 2009; Choy et al., 2011). In continuation, Liu et al. developed a similar Pd-catalyzed approach for the selective C3-acetoxylation of substituted indoles **43** with reduced catalyst loading (1.5 mol%) using PhI(OAc)_2_ **1** and KOH as a base (Scheme 4; Liu et al., 2011). Mechanistic insights revealed that the electrophilic palladation occurs at the C3 position of indole to generate Pd(II) species **45** which is oxidized to Pd(IV) intermediate **46** and subsequent reductive elimination affords corresponding C3-acetoxylated indoles **44**.

Acyloxylation of aliphatic sp^3^ C–H bond is another important method for regioselective C–O bond formation. Elegant protocols have been developed employing various nitrogen-based directing groups for the selective oxygenation of unactivated C(sp^3^)–H bond. In 2010, a novel chelation-assisted Pd-catalyzed C(sp^3^)–H acyloxylation of 8-methylquinolines in the presence of a stoichiometric amount of oxidant PhI(OAc)_2_ **1** was developed employing inexpensive arene carboxylic acids as acyloxy source (Zhang et al., 2010). A similar method for the catalytic acetoxylation of unactivated primary β-C(sp^3^)–H bond of *S*-methyl-*S*-2-pyridylsulfoximine-*N*-amides at room temperature was described by Sahoo's group (Rit et al., 2012). This method employs *S*-methyl-*S*-2-pyridylsulfoximine (MPyS) as a bidentate directing group and β-C–H acetoxylated products were synthesized using different carboxylic acids as solvent and acetate sources.

**Scheme 5:**
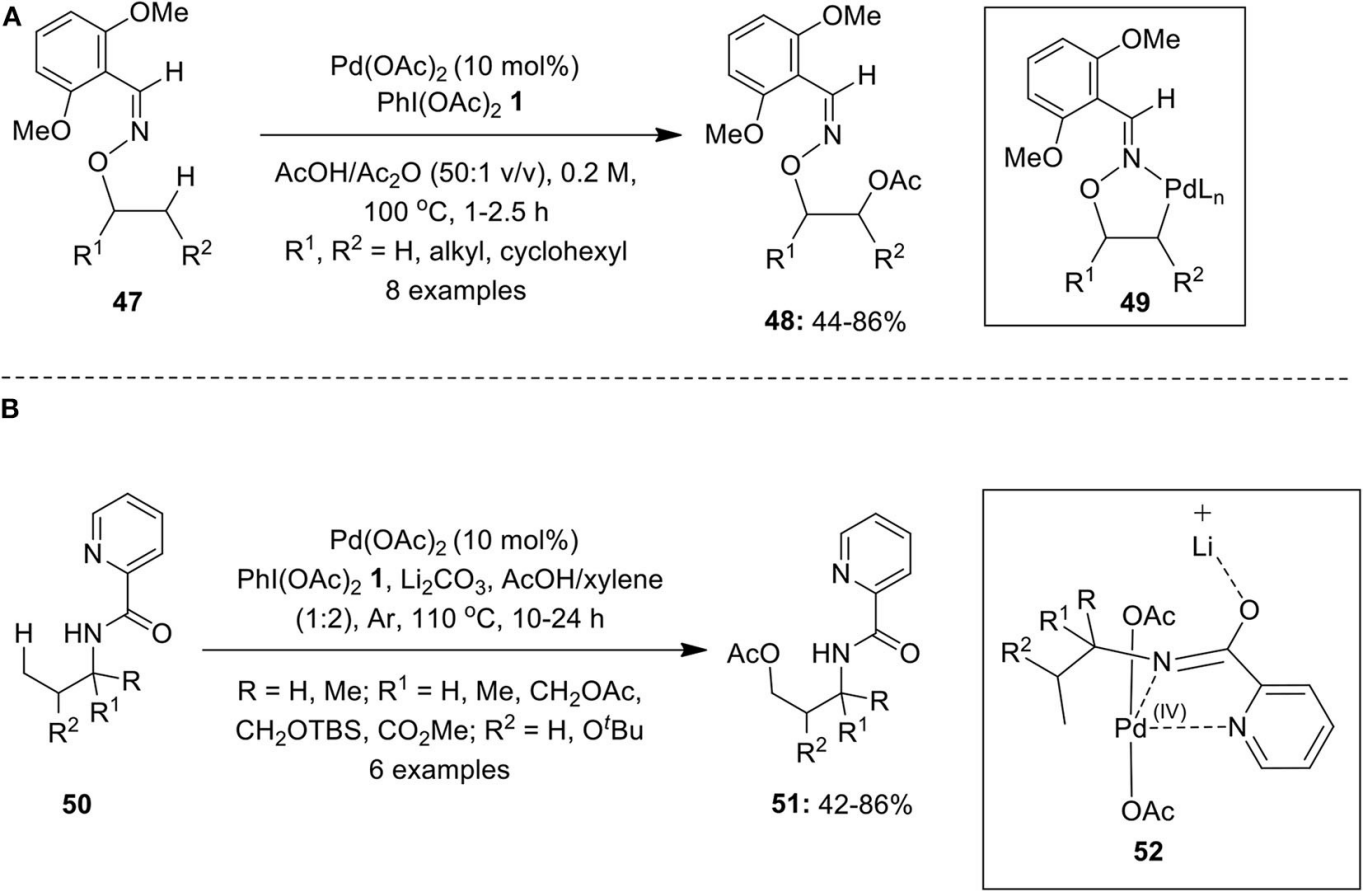
**(A)** Pd-catalyzed PIDA-mediated C(sp^3^)–H acetoxylation of substrates **47** and **(B)** Pd-catalyzed C(sp^3^)–H acetoxylation of alkylamines **50** using oxidant PIDA **1**.

Furthermore, a Pd-catalyzed oxime-directed β-C(sp^3^)–H acetoxylation of substrates **47** was performed employing PhI(OAc)_2_ **1** as a terminal oxidant (Ren et al., 2012). The catalytic reaction was expected to generate five-membered *exo*-palladacycle **49** which on oxidation gives masked 1,2-diols **48** (Scheme 5A). Also, the selective acetoxylation of β-methylene (CH_2_) and β-methine (CH) groups in cyclopentanol and norbornyl-derived alcohols were also carried out under the same reaction conditions. Furthermore, the deprotection of DG and acetyl group was carried out by using Zn/AcOH & K_2_CO_3_/MeOH, respectively, to yield diols in excellent yield. Later, Zhang's group described an elegant work on Pd(II)-catalyzed benzylic C(sp^3^)–H acetoxylation of picolinoyl- or quinoline-2-carbonyl-protected toluidine derivatives using PhI(OAc)_2_ **1** as an oxidant and acetate source (Ju et al., 2013).

In 2014, Li et al. performed a Pd(OAc)_2_-catalyzed acetoxylation of γ-C(sp^3^)–H bond of simple alkylamines **50** directed by picolinamide (PA) using oxidant PhI(OAc)_2_ **1** under an argon atmosphere (Li et al., 2014). The process provides an easy access to γ-acetoxylated product **51** in good yields (Scheme 5B). Additionally, C–H acetoxylation of γ-methyl group of arylamines proceeded smoothly under this condition. Authors speculated that reaction likely to procced via *C, N, N*-pincer type Pd(IV) intermediate **52**, in which Li_2_CO_3_ plays a crucial role as it interacts with *O*-imidate thereby suppressing the formation of cyclic azetidine through intramolecular C–H amination.

### C(sp^2^/sp^3^)–H Alkoxylation

Another interesting Pd-catalyzed transformation enabling C–O bond formation is the C–H alkoxylation of sp^2^ and sp^3^ bonds using hypervalent iodine reagents as oxidant. Chen and Shi's group independently reported two methods for the palladium-catalyzed PhI(OAc)_2_-mediated alkoxylation of methylene and methyl C(sp^3^)–H bonds with a range of aliphatic alcohols as alkoxylation reagents, using picolinamide (Zhang et al., 2012) and pyridine (Chen et al., 2013) as easily removable directing groups. Later, azo group-directed selective C(sp^2^)–H alkoxylation of azobenzene compounds with alcohols as the alkoxylation reagents was reported by Yin et al. using palladium catalysis in the presence of oxidant PhI(OAc)_2_ **1** (Yin et al., 2013). Additionally, Zhang and Sun demonstrated the regioselective alkoxylation of *ortho*-C(sp^2^)–H bond of 2-aryloxypyridines to provide *ortho*-alkoxylation products in the presence of catalytic amounts of Pd(OAc)_2_ and oxidant PhI(OAc)_2_ **1** (Zhang and Sun, 2014).

**Scheme 6:**
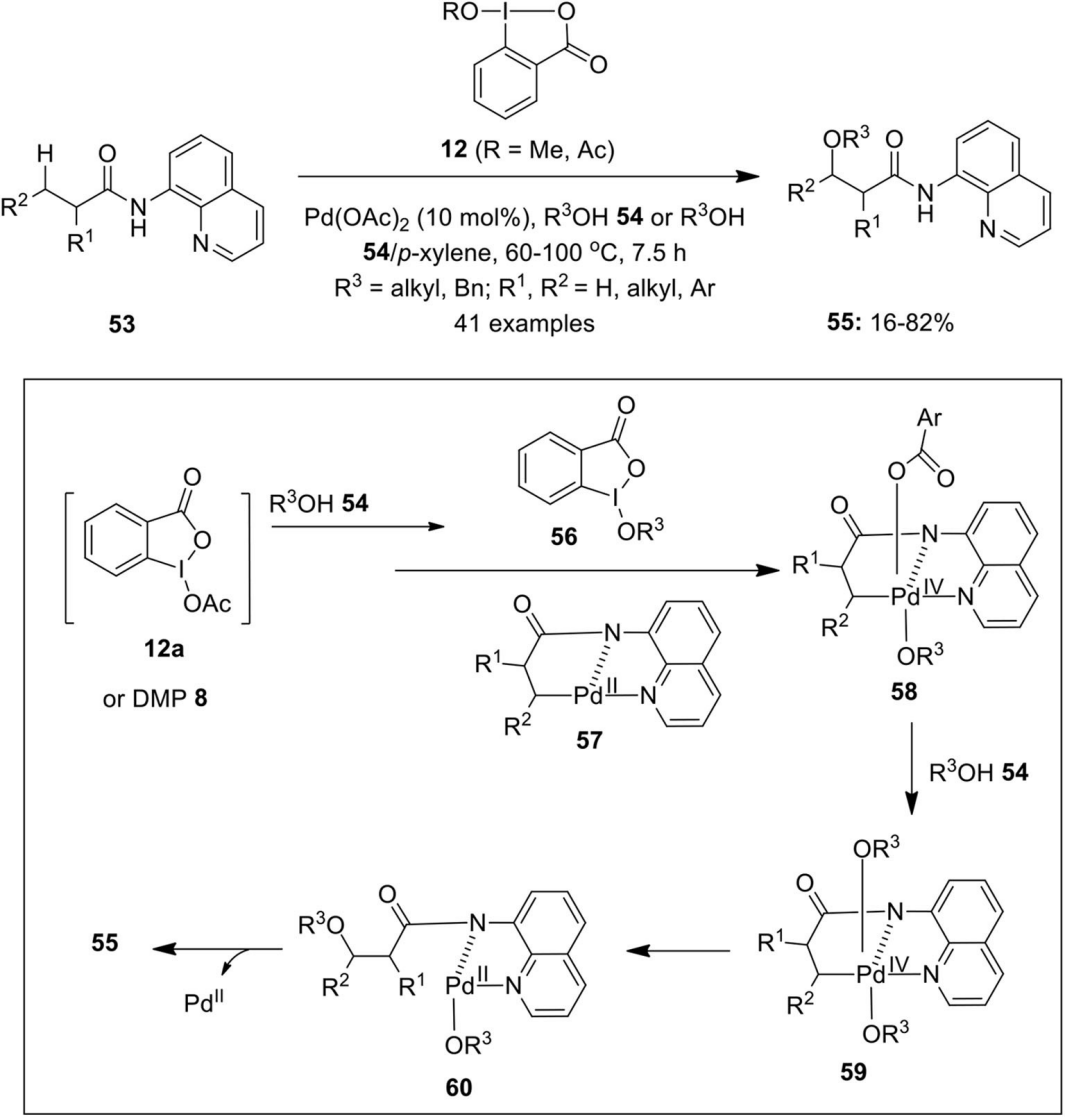
Pd-catalyzed C(sp^3^)–H bond alkoxylation of aminoquinoline-derived substrates **53** to provide β-alkoxylated products **55** using cyclic hypervalent iodine(III) reagent **12**.

Shan et al. for the first time employed cyclic hypervalent iodine(III) reagent **12** as an efficient oxidant in the Pd-catalyzed C(sp^3^)–H alkoxylation of unactivated methylene and methyl groups of 8-aminoquinoline-derived carboxylic acids **53** (Shan et al., 2013) (Scheme 6). The reaction worked perfectly well with DMP **8** (1.1 equivalents) at 110°C. Gratifyingly, C(sp^3^)-H alkoxylation of cyclic substrates such as cyclopentyl, cyclohexyl, and cycloheptyl were also perfomed under identical conditions. Furthermore, the synthetic applicability of present method was demonstrated for the alkoxylation of various analogs of Ibuprofen such as, Naproxen, Ketoprofen, and Flurbiprofen to afford alkoxylated products in variable yields. Based on preliminary mechanistic studies, authors proposed that either DMP **8** or 1-acetoxy-1,2-benziodoxole-3(1*H*)-one **12a** serve as key precursors for the *in situ* generation of cyclic iodine(III) oxidant **56** which oxidizes cyclopalladium(II) intermediate **57** to Pd(IV) intermediate **58**. Next, ArCO_2_ ligand of intermediate **58** could be easily displaced by alcohol **54** to give Pd(IV) intermediate **59** which finally undergoes C–O bond-forming reductive elimination to yield alkoxylated products **55** and regenerates palladium catalyst. Later, the same research group developed a similar method to prepare symmetrical and unsymmetric acetals via Pd-catalyzed regioselective double C(sp^3^)–H bond alkoxylation of 8-aminoquinoline-derived substrates using cyclic iodine(III) reagent **12** as an oxidant (Zong and Rao, 2014).

### C–H Oxidation and C–H Phosphorylation/Sulfonation

**Scheme 7:**
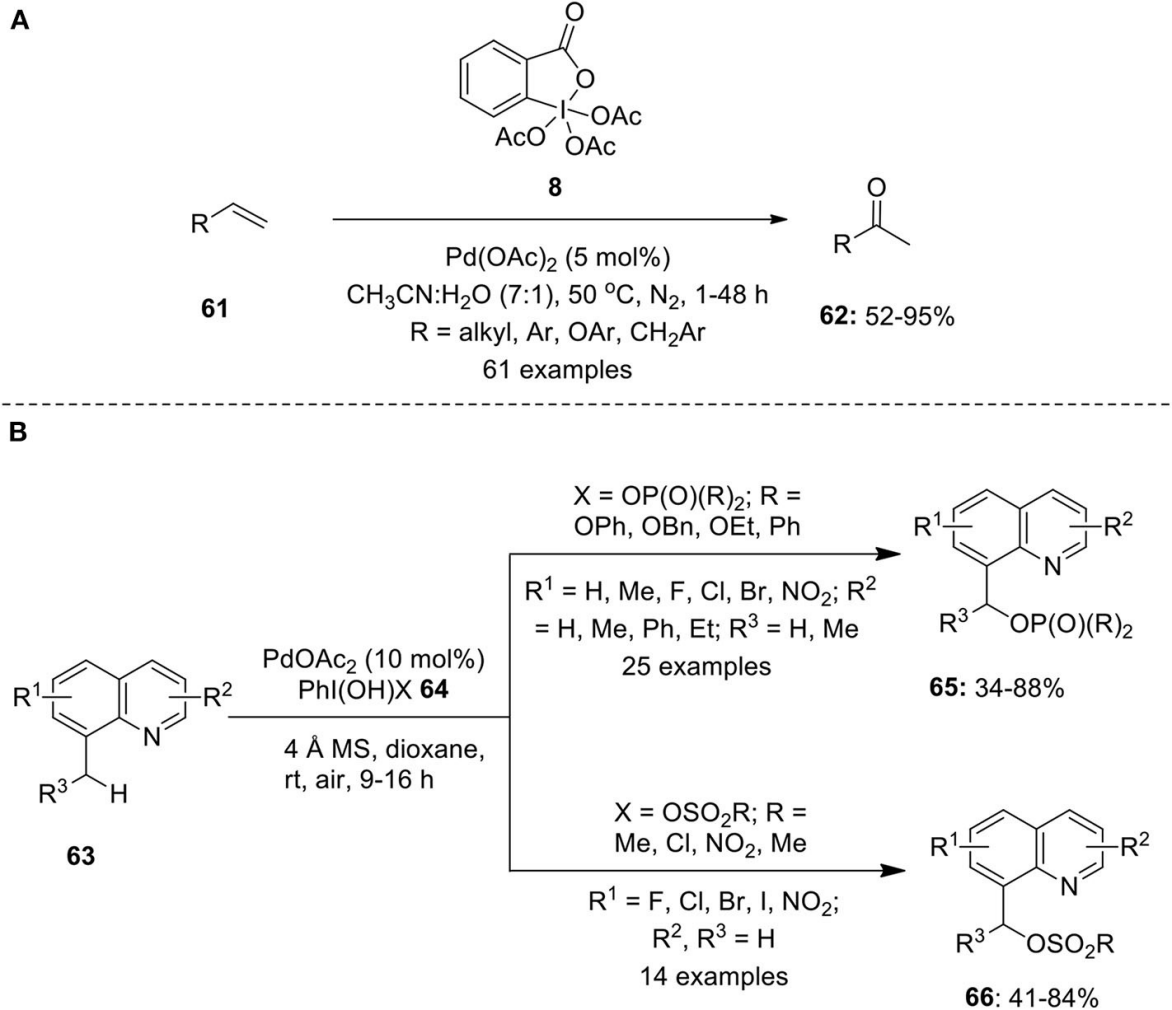
**(A)** Pd(II)-catalyzed C–H oxidation of terminal alkenes **61** using DMP **8** as an oxidant and **(B)** Pd(II)-catalyzed C–H phosphorylation/sulfonation of methylquinolines **63** using hypervalent iodine reagent **64**.

Chaudhari and Fernandes developed a Wacker–type oxidation protocol to convert various aliphatic and aromatic terminal alkenes **61** into functionally diversified methyl ketones 6**2** employing Dess-Martin Periodinane (DMP) **8** as an oxidant under nitrogen atmosphere (Chaudhari and Fernandes, 2016). Furthermore, the oxidation of a number of allylic or homoallylic compounds were also investigated under identical conditions to deliver methyl ketones in high yields. Excellent functional group compatibility, wide substrates scope and high isolated yields with complete Markovnikov selectivity are the key features associated with this methodology (Scheme 7A). A similar method for the conversion of terminal olefins into α,β-unsaturated ketones was developed by Bigi and White via a Wacker oxidation-dehydrogenation process employing Pd(CH_3_CN)_4_(BF_4_)_2_ (10 mol%) and PhI(OAc)_2_ **1** (25 mol%) co-catalytic system in the presence of 1,4-benzoquinone as oxidant (Bigi and White, 2013). Interestingly, PhI(OAc)_2_ **1** acts as a dehydrogenation catalyst and not as a terminal oxidant in this reaction.

Very recently, He et al. reported Pd(II)-catalyzed phosphorylation and sulfonation of unactivated benzyl C(sp^3^)–H bond of 8-methylquinolines **63** employing organophosphorus or sulfonate hypervalent iodine(III) reagents **64** as an oxidant and as a functional group source (He et al., 2019). Using this protocol, desired products **65** or **66** were obtained in moderate to high yields with broad substrates scope (Scheme 7B). Additionally, the scope of reaction was extended for the C(sp^2^)–H hydroxylation and arylation of 2-phenylpyridines.

Furthermore, limited protocols are available for the direct C–O bond formation without involving C–H bond functionalization. For instance, Kitamura and his research group disclosed efficient conversion of (trimethylsilyl)arenes into acetoxyarenes via Pd(OAc)_2_-catalyzed desilylative acyloxylation strategy using PhI(OCOCF_3_)_2_ **2** as an oxidant in AcOH (Gondo et al., 2015). Meanwhile, Cheng et al. reported Pd-catalyzed acetoxylative, hydroxylative and alkoxylative cycloisomerization of polysubstituted homoallenyl amides enabling preparation of functionalized 2-aminofurans using hypervalent iodine(III)reagents as oxidant (Cheng C. et al., 2015). Another striking example to prepare α-acetoxylated enones from propargylic substituted alkynes through Pd-catalyzed oxidative acetoxylation in the presence of terminal oxidant PhI(OAc)_2_ **1** and additive benzoquinone (10 mol%) in DMSO was developed (Jiang et al., 2016).

## C–C Bond Formation

Palladium-catalyzed C–H functionalization constitutes an important method in C–C bond formation reactions. A number of catalytic transformations have been developed using hypervalent iodine(III) reagents as oxidant.

### *via* Oxidative Cyclization

**Scheme 8:**
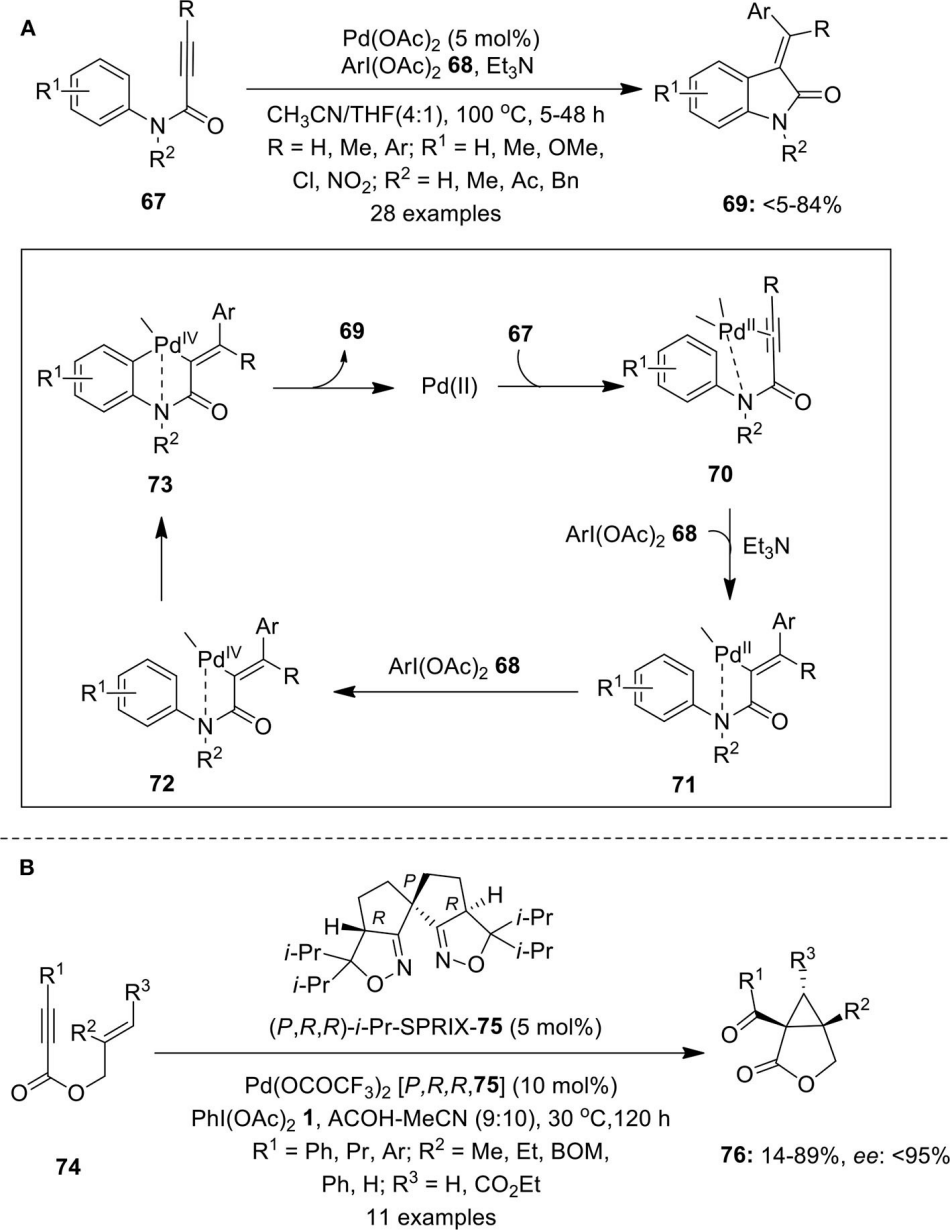
**(A)** Pd(II)-catalyzed synthesis of 3-(1-arylmethylene)oxindoles **69** using iodine(III) reagent **68** and **(B)** Pd(II)-catalyzed enantioselective synthesis of bicyclic lactones **76** by employing terminal oxidant PhI(OAc)_2_ **1**.

An interesting domino process highlighting Pd-catalyzed C–H functionalization of *N*-arylpropiolamides **67** using iodine(III) reagent **68** as an aryl source was demonstrated by Tang et al. (2008a). This elegant protocol leads to the synthesis of 3-(1-arylmethylene)oxindoles **69** in useful yields (Scheme 8A). The effect of various electron-rich and electron-deficient substituents on the aryl ring as well as on the triple bond was investigated. The catalytic cycle for this reaction starts with the coordination of Pd(II)-catalyst with alkyne and nitrogen to form intermediate **70** followed by cis-addition with Pd and ArI(OAc)_2_ **68** to give Pd(II) intermediate **71**. Oxidation of intermediate **71** by ArI(OAc)_2_ **68** forms Pd(IV) intermediate **72** which further gives intermediate **73** by activation of *ortho* C–H bond of **72**. Finally, intermediate **73** underwent reductive elimination to provide anticipated products **69** and releases active Pd(II) species. The same group also reported the synthesis of (*E*)-(2-oxindolin-3-ylidene)phthalimides and (*E*)-(2-oxoindolin-3-ylidene)methyl acetates via Pd-catalyzed C–H functionalization of *N*-arylpropiolamides using phthalimide and carboxylic acids as nucleophiles, respectively (Tang et al., 2008b,c). Later, synthesis of CF_3_-substituted oxindoles was accomplished through palladium-catalyzed PhI(OAc)_2_-mediated intramolecular trifluoromethylation of *N*-alkyl-*N*-arylacrylamide derivatives using TMSCF_3_ as an efficient trifluoromethyl source in the presence of Lewis acid Yb(OTf)_3_ (Mu et al., 2012).

Tong et al. coined the first Pd-catalyzed oxidative cyclization of 1,6-enynes into the corresponding bicyclo[3.1.0]hexane derivatives using oxidant PIDA **1** (Tong et al., 2007). Later, Sanford and Tong's research groups independently developed similar pathways toward the synthesis of multi-substituted bicyclo[3.1.0] ring systems using bipyridine as ligand (Welbes et al., 2007; Liu et al., 2008; Lyons and Sanford, 2009). Furthermore, Tsujihara et al. developed the first example featuring asymmetric Pd(II)/Pd(IV) catalysis for the enantioselective synthesis of bicyclic lactones **76** from 1,6-enynes **74** in variable yields with up to 95% enantiomeric excess (Scheme 8B) (Tsujihara et al., 2009). Reaction utilizes the spiro bis(isoxazoline) 7**5** (abbreviated as SPRIXs) as chiral ligand, Pd-SPRIX **75** complex as catalyst and PhI(OAc)_2_ **1** as terminal oxidant.

**Scheme 9:**
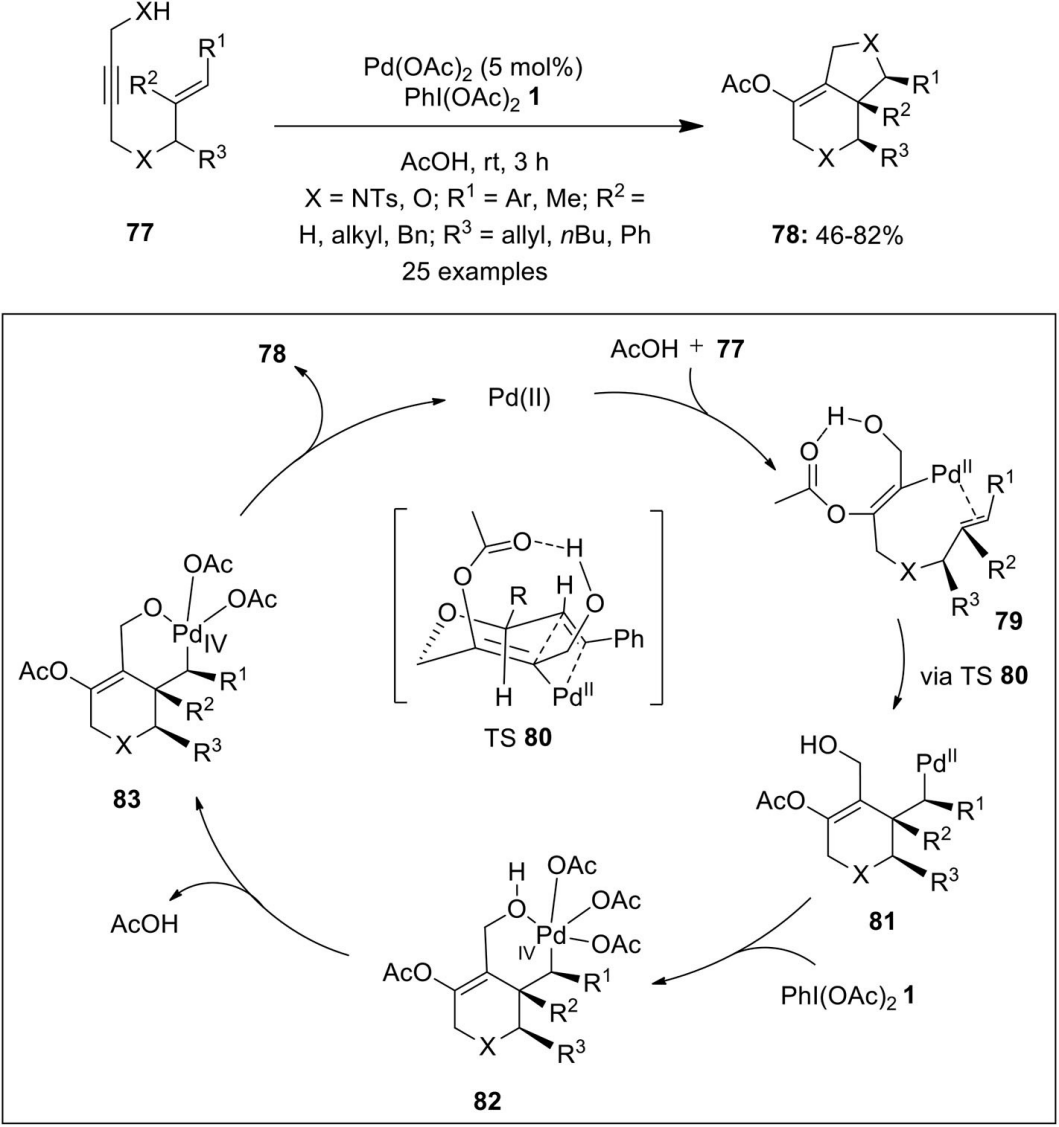
Pd(II)-catalyzed synthesis of bicyclic heterocycles **78** from 1,6-enynes **77** using oxidant PhI(OAc)_2_ **1**.

Very recently, Xu et al. published an excellent example on the Pd(II/IV)-catalyzed intramolecular cycloaddition of the propargylic alcohol/amine and alkene of substrates **77** via acetoxylative (3 + 2) annulation strategy enabling the synthesis of bicyclic heterocycles **78** in significant yields (Xu et al., 2019) (Scheme 9). The possible catalytic cycle for this oxidative cycloaddition reaction initiates through the acetoxypalladation process generating alkenyl-Pd(II) intermediate **79** which is subsequently transform into alkyl-Pd(II) intermediate **81** via chair-like transition state (TS) **80**. Next, PhI(OAc)_2_-mediated oxidation gives bicyclic Pd(IV) intermediate **82** which further gives **83** through loss of a molecule of AcOH. Finally, intermediate **83** undergoes direct C–O reductive elimination to deliver product **78** and regenerate palladium catalyst to continue the catalytic cycle (Scheme 9).

### *via* C–H Bond Arylation

**Scheme 10:**
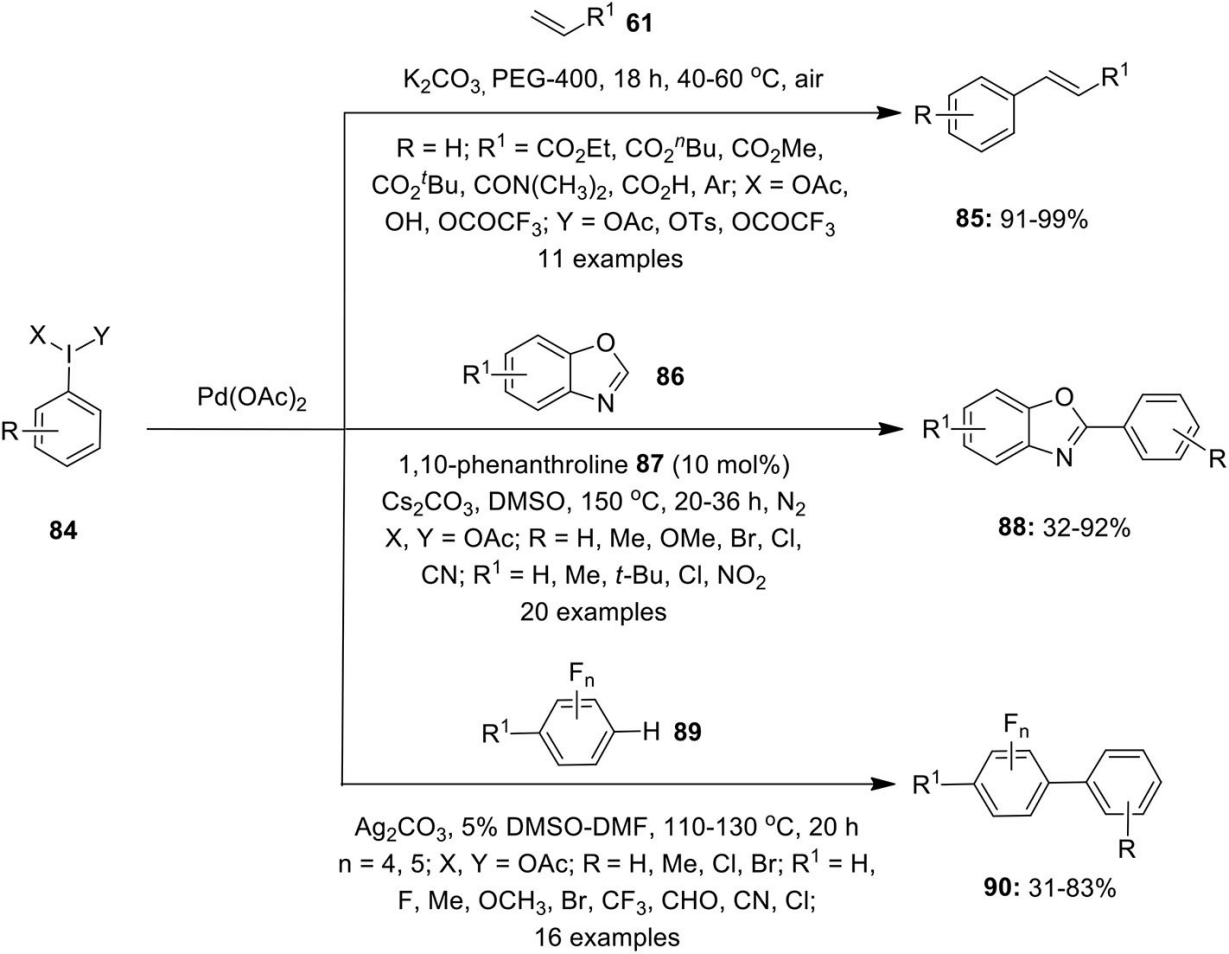
Pd(II)-catalyzed Hseck-type coupling reactions using hypervalent iodine(III) reagents **84**.

In 2011, Qu et al. demonstrated a Heck-type coupling reaction between olefins **61** and iodobenzene generated *in situ* from hypervalent iodine reagents **84** (Qu et al., 2011). The coupling reaction was performed using Pd(OAc)_2_ (4 mol%), base K_2_CO_3_, and PEG-400 as solvent media under an open atmosphere at 40–60°C to afford coupling products **85** in 91–99% yields (Scheme 10). The catalytic system was free from any ligand or additive and possesses an excellent recyclability of catalyst (Scheme 10). A similar Pd-catalyzed Heck-type arylation of terminal alkenes with aryliodine(III) diacetates was developed by Evdokimov et al. (2011).

Later, Cheng's research group performed Pd(II)-catalyzed C–H functionalization of benzoxazoles **86** to furnish arylation products **88** using iodobenzene diacetates **84** as arylating reagent in the presence of ligand 1,10-phenanthroline **87** and base Cs_2_CO_3_ (Yu et al., 2012) (Scheme 10). Similarly, Fu et al. employed aryliodine(III) diacetates **84** as a coupling partner in the Pd-catalyzed C–H arylation of polyfluoroarenes **89** (Fu et al., 2015). Detailed mechanistic studies revealed *in situ* generation of aryliodides by the reduction of ArI(OAc)_2_ **84** under basic conditions to furnish desired polyfluorobiaryls products **90** in moderate to good yields (Scheme 10).

Another interesting strategy to establish C–C bond formation is Pd-catalyzed oxidative coupling reactions of aromatic halides with different arylation reagents. Considering this, Xiong et al. illustrated the synthesis of useful symmetrical biaryls employing aryliodine(III) diacetates **68** as aryl precursor via Pd-catalyzed homocoupling reaction (Xiong et al., 2015). The reaction was carried out in DMF at 110°C in the presence of Pd(OAc)_2_ (10 mol %), K_2_CO_3_ (4 equiv.) under an air atmosphere. In 2016, Wang et al. reported ligand-free arylation of pyridines and quinolines via direct coupling of bromo-substituted pyridines or quinolines with hypervalent iodine(III) compounds as efficient arylating reagents in the presence of catalyst PdCl_2_ and base Cs_2_CO_3_ in DMF at 110°C (Wang et al., 2016).

### *via* C–H Trifluoromethylation/Alkynylation

Mu et al. reported Pd-catalyzed C–H trifluoromethylation to prepare C2-trifluoromethylated indoles employing Ruppert–Prakash reagent, TMSCF_3_ as CF_3_ source in presence of oxidant PhI(OAc)_2_ **1** and base CsF at room temperature (Mu et al., 2011). In 2013, Tolnai et al. employed 2 mol% of Pd(MeCN)_4_(BF_4_)_2_ catalyst in the regioselective C2-alkynylation of *N*-alkylated indoles using 1-[(Triisopropyllsilyl)ethynyl]-1,2-benziodoxol-3(1*H*)-one (TIPS-EBX) as an alkynylating reagent (Tolnai et al., 2013). This reaction gave direct access to substituted 1-alkylated-2-[(triisopropylsilyl)ethynyl]-1*H*-indole at ambient temperature under air atmosphere in significant yields. A number of substituents including Cl, Br, F, & I remained intact in the final products which could be used for further synthetic modifications.

## C–N Bond Formation

**Scheme 11:**
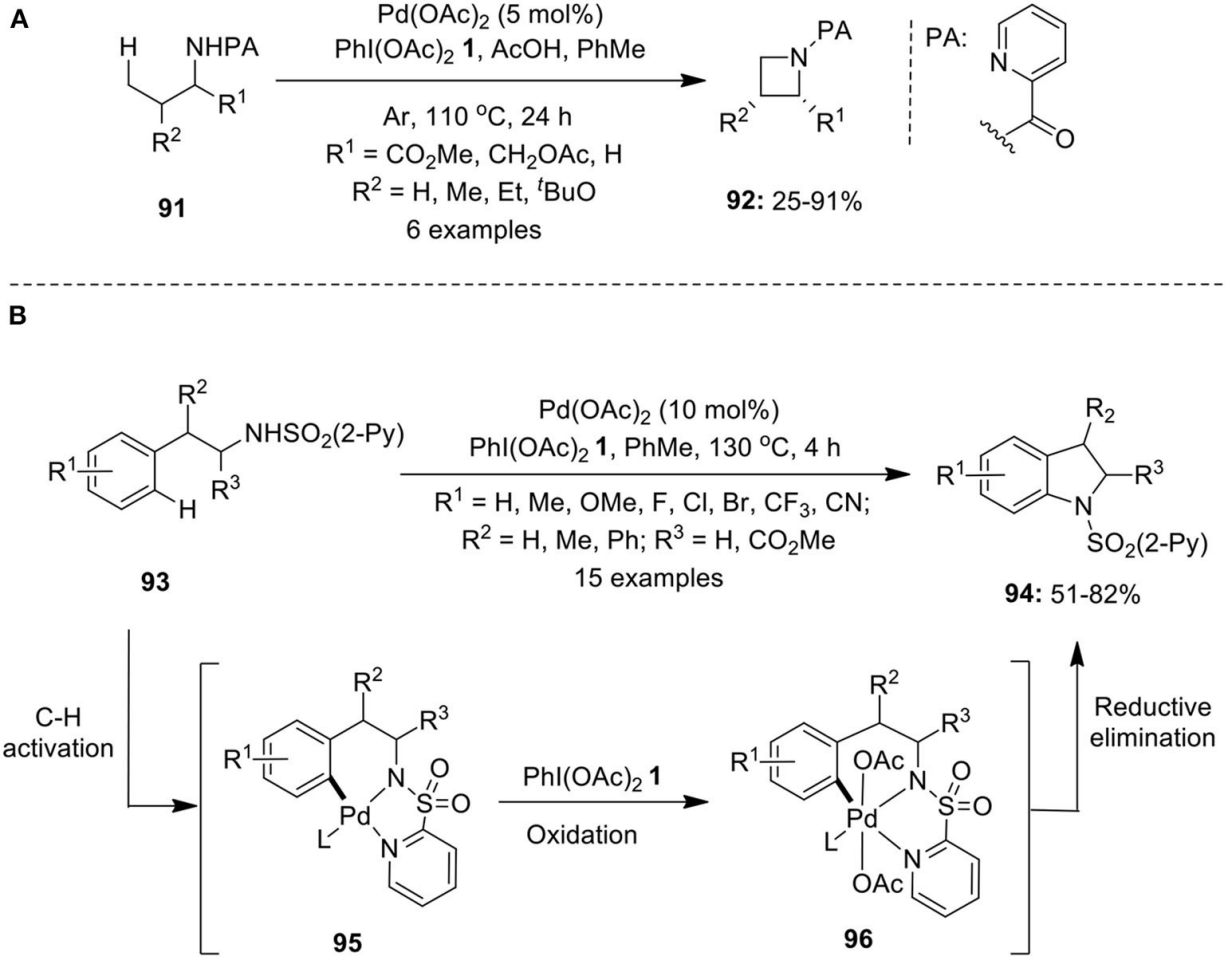
**(A)** Pd(OAc)_2_-catalyzed synthesis of azetidines **92** using oxidant PhI(OAc)_2_ **1** and **(B)** Pd(OAc)_2_-catalyzed synthesis of indolines **94** using oxidant PhI(OAc)_2_ **1**.

Catalytic C–H activation/C–N bond formation has gained considerable attention in recent times as it represents the versatile approach for the construction of *N*-containing aliphatic/aromatic heterocycles. Several intra- and intermolecular protocols for the palladium-catalyzed amination of sp^3^ and sp^2^ C–H bonds have been developed using hypervalent iodine reagents as oxidant. Gaunt has disclosed an elegant work on intramolecular C–H amination of *N-*substituted biphenyls for the synthesis of carbazoles at room temperature using catalyst Pd(OAc)_2_ and oxidant PhI(OAc)_2_ **1** in the presence of acetic acid in toluene (Jordan-Hore et al., 2008). Furthemore, Chen's research group synthesized azetidines **92** via intramolecular C–H amination of picolinamide-directed amine substrates **91** employing Pd(OAc)_2_ and PhI(OAc)_2_ **1** in toluene under argon atmosphere (Scheme 11A; He et al., 2012a). Additionally, the synthesis of pyrrolidines was also accomplished under identical reaction conditions.

Mei et al. demonstrated the synthesis of indolines **94** via Pd(OAc)_2_-catalyzed intramolecular C–H activation/C–N cyclization of phenethylamine derivatives **93** using 2-pyridinesulfonyl as efficient directing group in the presence of oxidant PhI(OAc)_2_ **1** (Mei et al., 2013). The intramolecular amination proposed to proceed via Pd-catalyzed C–H activation to generate organopalladium(II) complex **95**, followed by oxidation to form palladium(IV) intermediate **96** and final C–N reductive elimination to provide anticipated products **94**. Furthermore, the 2-pyridinesulfonyl moiety was easily removed under mild reaction conditions by treating with magnesium in MeOH at 0^o^C (Scheme 11B). Similar protocols to prepare indolines were independently reported by Daugulis, Chen, and Shi's research groups employing Pd(OAc)_2_/PhI(OAc)_2_ **1** catalytic system (He et al., 2012b; Nadres and Daugulis, 2012; Ye et al., 2013).

On the other hand, Yin et al. disclosed an intermolecular allylic C–H amination of alkyl olefins to furnish linear (*E*)-allylimides using *O*-alkyl *N*-sulfonylcarbamates as nitrogen nucleophile. The catalytic system comprises of catalyst Pd(OAc)_2_ (5 mol%), terminal oxidant PhI(OPiv)_2_, additive 1,4-naphthoquinone (20 mol%) and base Bu_4_NOAc (Yin et al., 2010). Another interesting protocol featuring Pd-catalyzed regioselective intermolecular C–H amination of multi-substituted arenes using phthalimide as the nitrogen source was developed by Hartwig and co-workers (Shrestha et al., 2013).

## C–B and C–SI Bond Formation

**Scheme 12:**
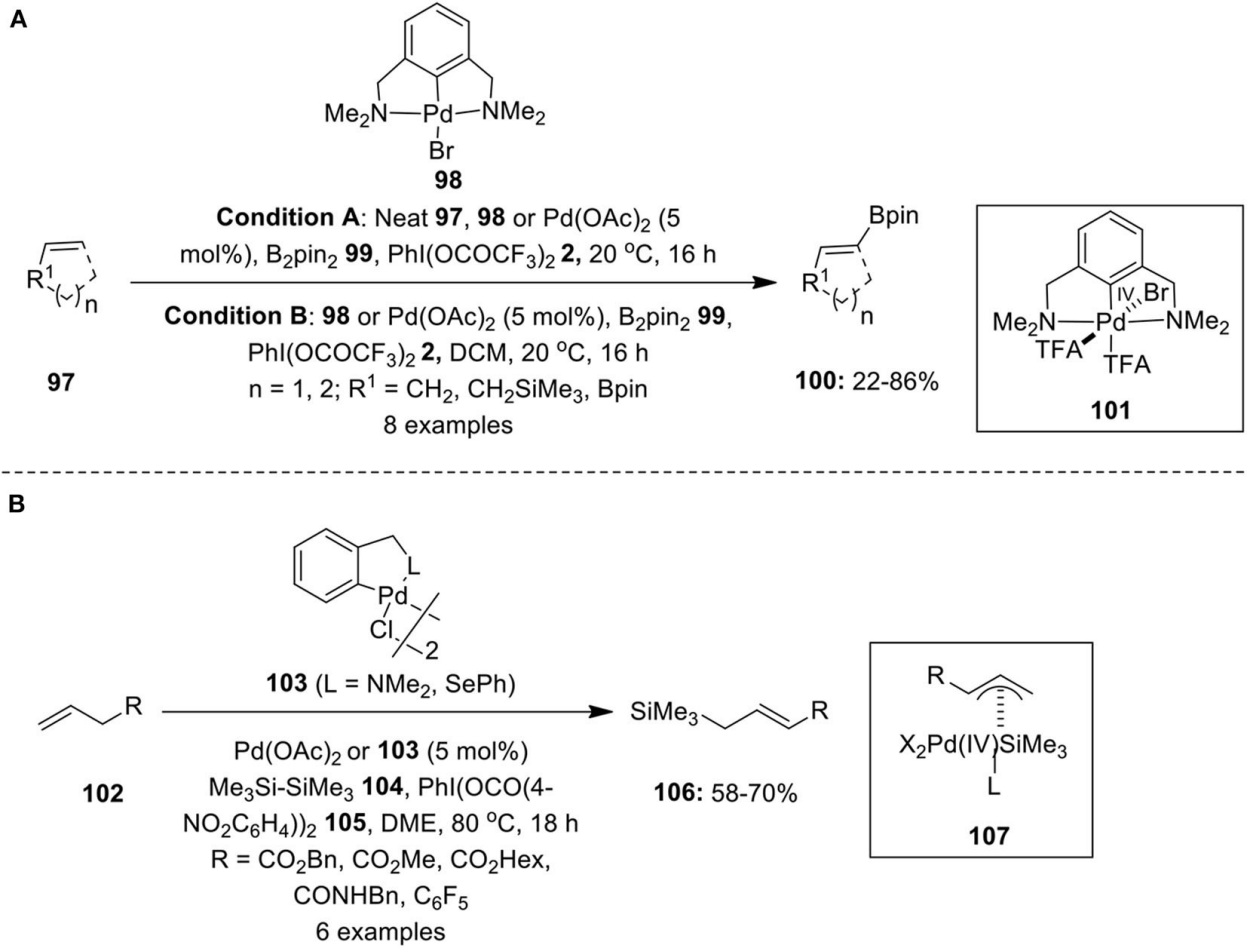
**(A)** Palladium-catalyzed C–H borylation of olefins **97** mediated by PhI(TFA)_2_ **2** and **(B)** Palladium-catalyzed C–H silylation of terminal olefins **102** using PhI(OCO(4-NO_2_C_6_H_4_))_2_ **105** as an oxidant.

Sazabo and his research group demonstrated the first example of selective C–H borylation of simple alkenes **97** employing a palladium pincer complex **98** as an effective catalyst and PhI(TFA)_2_ **2** as an essential oxidant (Selander et al., 2010). Reaction was performed in neat alkenes (Condition A) or diluted alkenes (Condition B) at ambient temperature (Scheme 12A). Simple cycloalkenes **97** such as cyclopentene and cyclohexene (R^1^: CH_2_; *n* = 1, 2) were readily borylated with B_2_pin_2_ **99** to provide valuable organoboronates **100** with excellent vinylic selectivity. However, in the case that cycloheptane selectivity was reversed, the formation of allylic product was favored. Further borylation reaction of acyclic alkenes such as allylsilane and vinylboronate **98** (R^1^: CH_2_SiMe_3_, Bpin; *n* = 0) were also carried out under similar reaction conditions. Additionally, the course of reaction was also studied with Pd(OAc)_2_ as a catalyst, but products were obtained in albeit lower yields. The key step involves formation of Pd(IV) intermediate **101** via oxidation of catalyst using PhI(TFA)_2_ **2**, which further undergoes transmetalation with B_2_pin_2_ and subsequent reaction with alkenes to deliver desired products **100**. Furthermore, the borylation reaction proceeded with excellent vinylic selectivity, except in the case of cycloheptane which yielded allylic products preferentially.

Later, the same research group developed the first Pd-catalyzed oxidative allylic C–H silylation of terminal olefins **102** using hexamethyldisilane **104** as the silyl source (Larsson et al., 2011). This catalytic process employs hypervalent iodine(III) reagent PhI(OCO(4-NO_2_C_6_H_4_))_2_ **105** as an oxidant and Pd(OAc)_2_ or nitrogen and selenium-based palladium catalyst **103** (Scheme 12B). The reaction follows Pd(II)/Pd(IV) pathway involving formation of allylpalladium complex **107** which presumably undergoes reductive elimination to furnish allylsilanes **106**. Moreover, the functional groups such as ester, benzyl, and amide were well-tolerated under the oxidizing conditions and anticipated products **106** were obtained with high regio- and stereoselectivity.

## C–Halogen Bond Formation

**Scheme 13:**
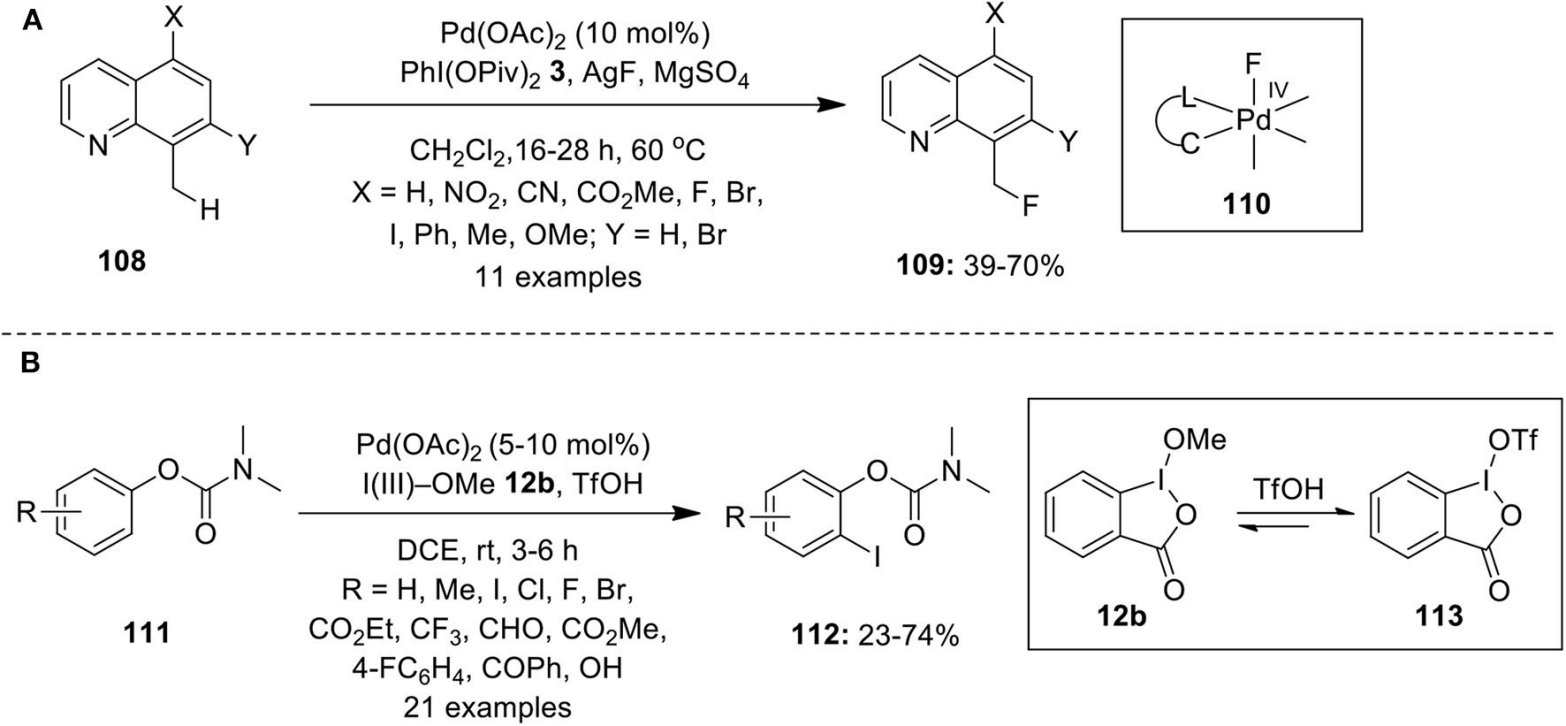
**(A)** Pd(II)-catalyzed C–H fluorination of 8-methylquinoline analogs **108** using oxidant PhI(OPiv)_2_ **3** and **(B)** Pd(II)-catalyzed C–H iodination of phenol carbamates **111** by employing Togni's reagent **12b**.

Palladium-catalyzed conversion of C–H bond into C–Halogen bond is an attractive method to access valuable aryl halides. However, in the past limited studies have been carried out in C–H halogenation reactions via high valent palladium catalysis. McMurtrey et al. published the first report on Pd-catalyzed C–H fluorination employing AgF as a fluoride source in combination with iodosobenzene dipivalate, PhI(OPiv)_2_ **3** as oxidant (McMurtrey et al., 2012). Although the detailed mechanism is not given, high valent Pd-alkyl fluoride complex **110** was hypothesized as the key intermediate in this process that would further undergo C–F bond-forming reductive elimination to yield fluorination products **109** in moderate to good yields (Scheme 13A). Furthermore, the substrates **108** bearing electron-withdrawing groups produced better results than those with electron-donating groups.

In 2015, Rao's research group achieved *ortho* C–H iodination of phenol carbamates **111** through palladium catalysis employing Togni's reagent **12b** as an iodine source and as oxidant in DCE/TfOH at room temperature (Sun et al., 2015). Both electron-rich and -deficient substituents were well-tolerated, leading to the preparation of *o*-iodinated phenol derivatives in fair to good yields (Scheme 13B). NMR studies revealed that some part of cyclic iodine(III)reagent on treatment with TfOH gets converted into **113** and probably both oxidants participates in this reaction.

## Alkene Difunctionalization

**Scheme 14:**
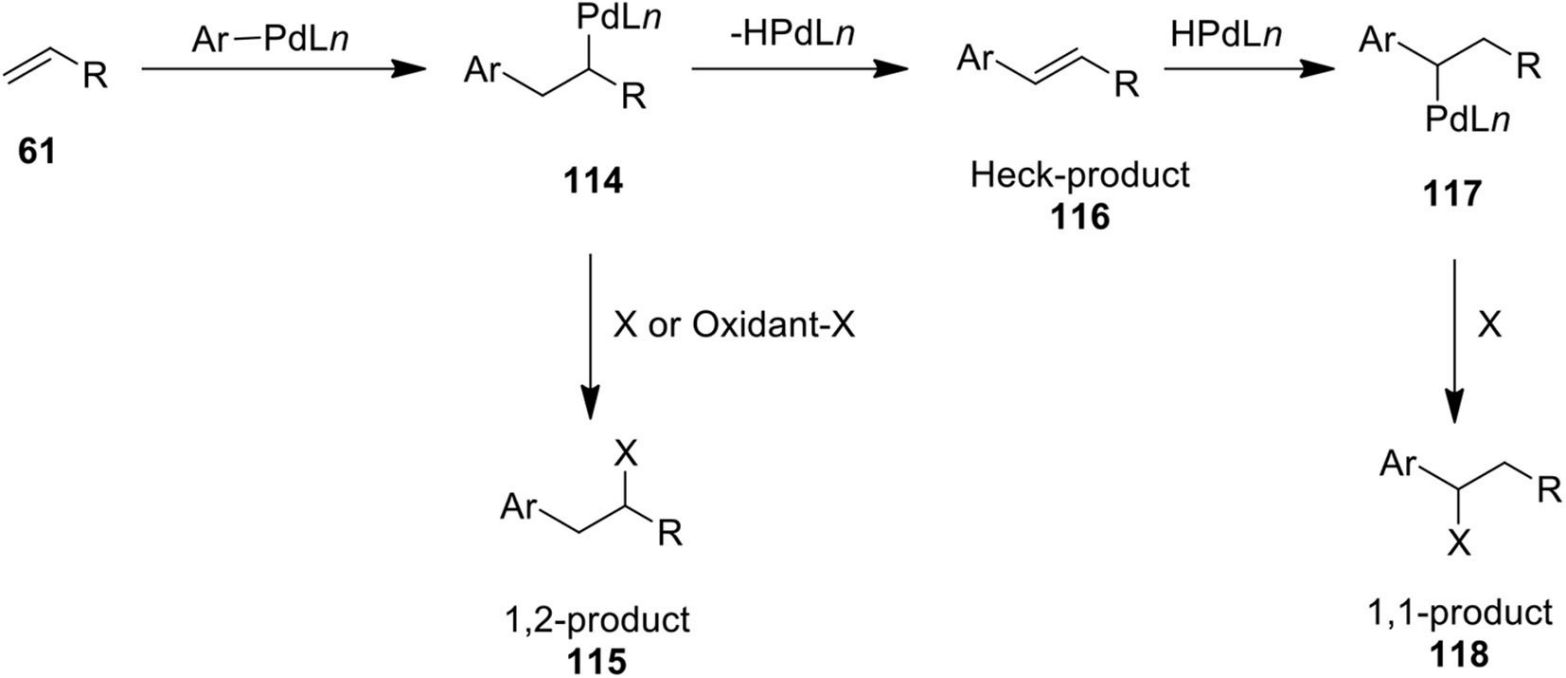
Plausible mechanism for the Pd(II)-catalyzed difunctionalization of alkenes **61**.

Palladium-catalyzed difunctionalization of simple alkenes using hypervalent iodine reagents has emerged as a powerful method in organic synthesis. Various 1,1- and 1,2-difunctionalization protocols have been developed by several researchers for preparing a diverse array of useful molecules from alkenes. A general mechanism for the Pd-catalyzed oxidative functionalization of alkenes **61** is shown in Scheme 14. These transformations proposed to proceed through the formation of δ-alkyl Pd^II^ intermediate **114** obtained via olefin insertion into the aryl-Pd bond. Next, Heck intermediate **114** undergoes β-hydride elimination to form Heck product **116** and the resulting -HPdL*n*-species readds again to give benzylic Pd(II) intermediate **117** which can be intercepted with suitable nucleophile to furnish 1,1-difunctionalized product **118**. Furthermore, Heck intermediates **114** could be oxidatively functionalized into 1,2-difunctionalized product **115** in the presence of suitable nucleophile or oxidant.

### Pd(II)-Catalyzed 1,1-Difunctionalization of Alkenes

**Scheme 15:**
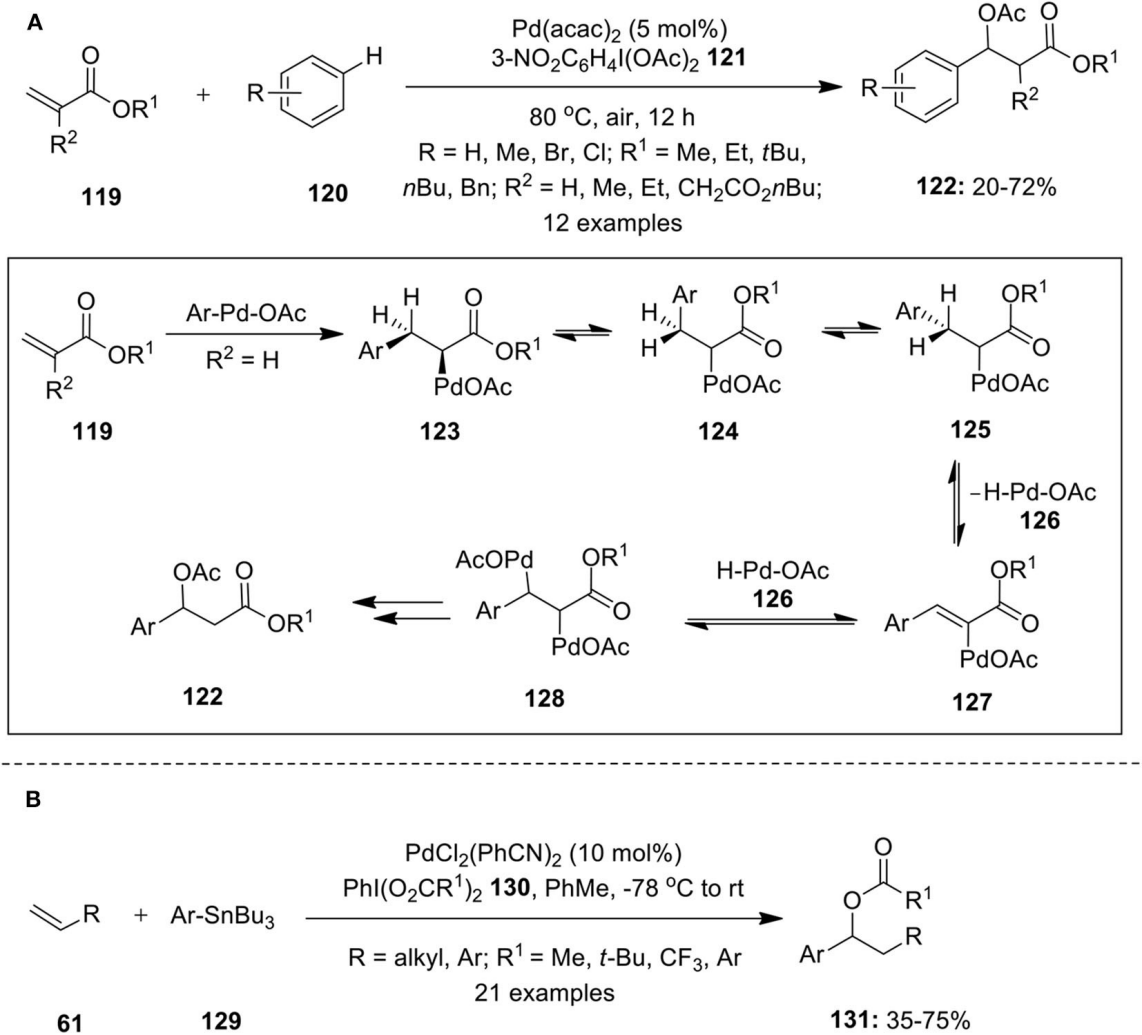
**(A)** Pd(II)-catalyzed 1,1-difuntionalization of acrylate derivatives **119** using iodine(III) reagent **121** and **(B)** Pd(II)-catalyzed 1,1-difuntionalization of terminal olefins **61** using iodine(III) reagent **130** as oxidant.

Pd-catalyzed hypervalent iodine mediated 1,1-difunctionalization of alkenes are rare and only few examples are available in the literature. Moran and co-author published an article highlighting 1,1-difuntionalization of acrylate derivatives **119** using palladium catalysis (Rodriguez and Moran, 2009). This reaction involves three component coupling of activated alkenes **119**, substituted arenes **120** and hypervalent iodine(III) reagent **121** in acetic acid. Reaction possibly involves formation of Heck intermediate **123** via Heck-type addition of aryl-Pd complex to the alkene **119**, followed by β-hydride elimination and subsequent readdition of Pd-H species **126** at benzylic site to form intermediate **128**. Finally, oxidation of **128** to Pd(IV) intermediate using iodine(III) reagent **121** and subsequent functionalization with acetate ion lead to the formation of desired 1,1-aryloxygenated compounds **122** (Scheme 15A).

Later, Satterfield et al. disclosed 1,1-aryloxygenation protocol wherein arylstannanes **129** were successfully coupled with terminal olefins **61** in the presence of hypervalent iodine reagents (PhI(O_2_CR^1^)_2_) **130** as an oxidant and palladium catalyst, PdCl_2_(PhCN)_2_ (Satterfield et al., 2011). This catalytic approach enabled simultaneous generation of C–C and C–O bond in a single step furnishing 1,1-arylacetoxylated products **131** in significant yields (Scheme 15B).

### 1,2-Difunctionalization of Alkenes

Over the years, great progress has been achieved in the field of Pd-catalyzed 1,2-difunctionalization of olefins using various hypervalent iodine reagents. Based on this strategy, a number intra- and intermolecular synthetic transformations such as diamination, dioxygenation, aminoacetoxylation, fluoroamination, and oxidative amination have been developed.

#### Intramolecular 1,2-Difunctionalization of Alkenes

Catalytic intramolecular difunctionalization of alkenes is one of the most powerful methods for the construction of heteroatom containing aromatic and aliphatic cyclic compounds. Sanford and co-workers reported Pd-catalyzed intramolecular oxidative amination strategy for the synthesis of tetrahydrofurans using oxidant PIDA **2** (Desai and Sanford, 2007). Subsequently, Muniz's research group developed an intramolecular alkene diamination protocol for the preparation of bisindoline and cyclic urea scaffolds using palladium catalysis (Streuff et al., 2005; Muniz, 2007; Muniz et al., 2008). Furthermore, Wu et al. performed Pd-catalyzed intramolecular aminofluorination of unactivated *N*-tosylamine alkenes with AgF as fluorinating reagent in the presence of PhI(OCO*t*Bu)_2_ as terminal oxidant and MgSO_4_ as additive in acetonitrile at room temperature (Wu et al., 2009).

Zhu's research group reported domino carboacetoxylation of *N*-aryl acrylamides to synthesize 3,3′-disubstituted oxindoles and spiropyrrolidinyloxindoles using catalytic amount of Pd(OAc)_2_ or PdCl_2_ and oxidant PhI(OAc)_2_ **1** in AcOH at 100°C (Jaegli et al., 2010). Furthermore, another intramolecular carboacetoxylation protocol for the preparation of acetoxymethyl-substituted cyclopentane derivatives via oxidative cyclization of 4-pentenyl-substituted malonate esters employing bis(acetonitrile)dichloropalladium catalyst and oxidant PhI(OAc)_2_ **1** was developed (Fujino et al., 2010). The reaction was performed in the presence of base titanium tetraisopropoxide in DCE/Ac_2_O (1:1) solvent system at 50°C.

**Scheme 16:**
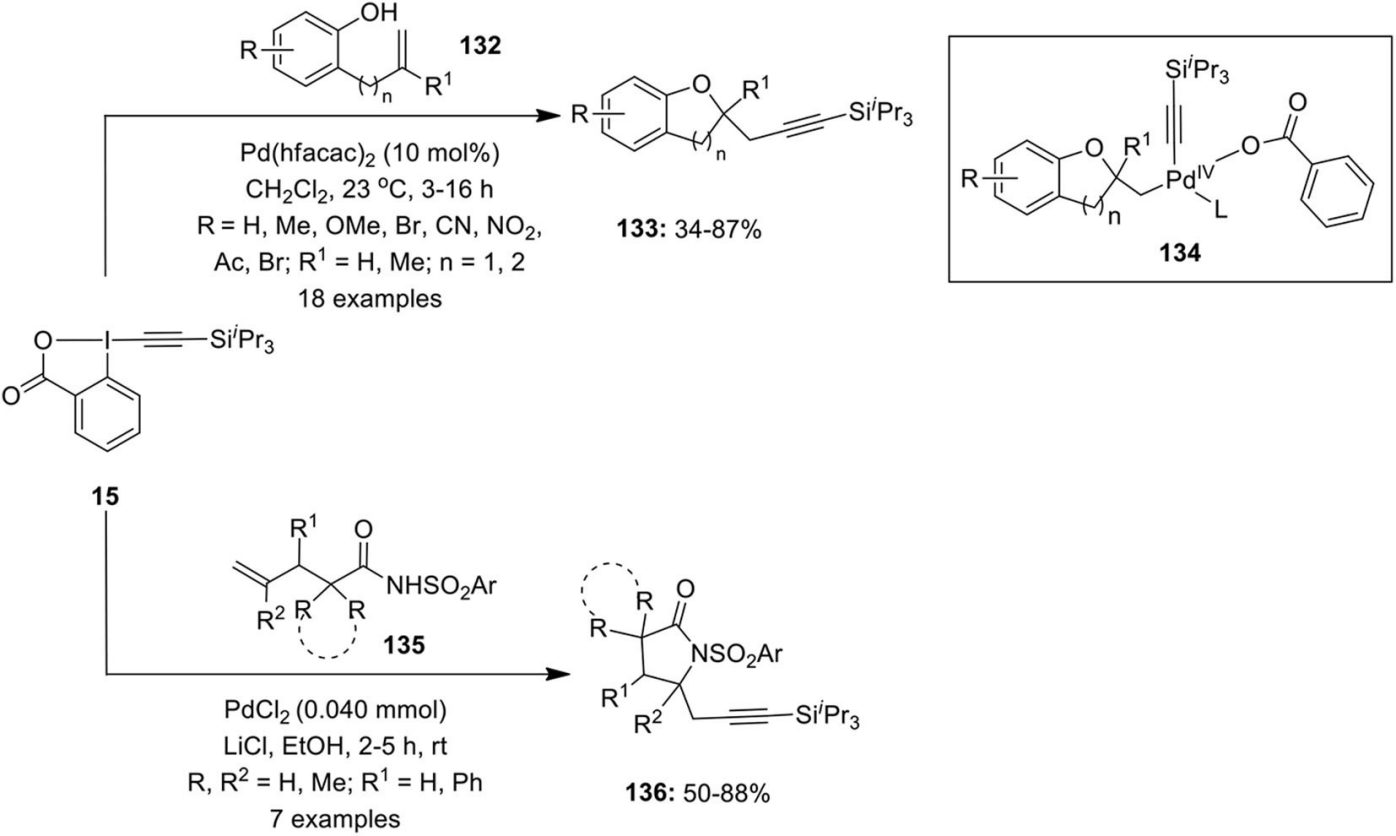
Pd-catalyzed intramolecular oxyalkynylation of phenols **132** and aminoalkynylation of activated amides **135** by using TIPS-EBX **15** as acetylene transfer reagent.

In 2010, Nicolai et al. reported the first intramolecular oxyalkynylation of non-activated terminal alkenes **132** using 1-[(triisopropylsilyl)ethynyl]-1,2-benziodoxol-3(1*H*)-one (TIPS-EBX) **15** as acetylene transfer reagent in DCM in the presence of 10 mol% of Pd(hfacac)_2_ as an efficient Pd species (Nicolai et al., 2010). The reaction was successful with different phenol substrates **132** giving cyclic ethers **133** via Pd(IV) intermediate **134** in variable yields (Scheme 16). Additionally, the scope of the reaction was examined with both aromatic and aliphatic carboxylic acids leading to the synthesis of γ-lactones under the same optimized reaction condition.

In continuation with the difunctionalization reaction, the Waser's group described an intramolecular aminoalkynylation of activated olefins **135** to afford 4-propargyl lactams **136** employing TIPS-EBX **15** as alkynylating agent (Nicolai et al., 2011). In the presence of PdCl_2_ and LiCl, the active catalyst lithium palladate, Li_2_[PdCl_4_] generates *in situ* which catalyzes the carboamination reaction (Scheme 16). Furthermore, the present protocol was successfully utilized for the synthesis of 4-propargyl oxazolidinone and imidazolidinones through the cyclization of allyl carbamates and allyl ureas, respectively. Additionally, the synthesized γ- and δ-lactams were employed as precursors for the synthesis of bicyclic heterocycles pyrrolizidine and indolizidine and also in the total synthesis of natural product (±)-trachelanthamidine (Nicolai et al., 2011).

#### Intermolecular 1,2-Difunctionalization of Alkenes

**Scheme 17:**
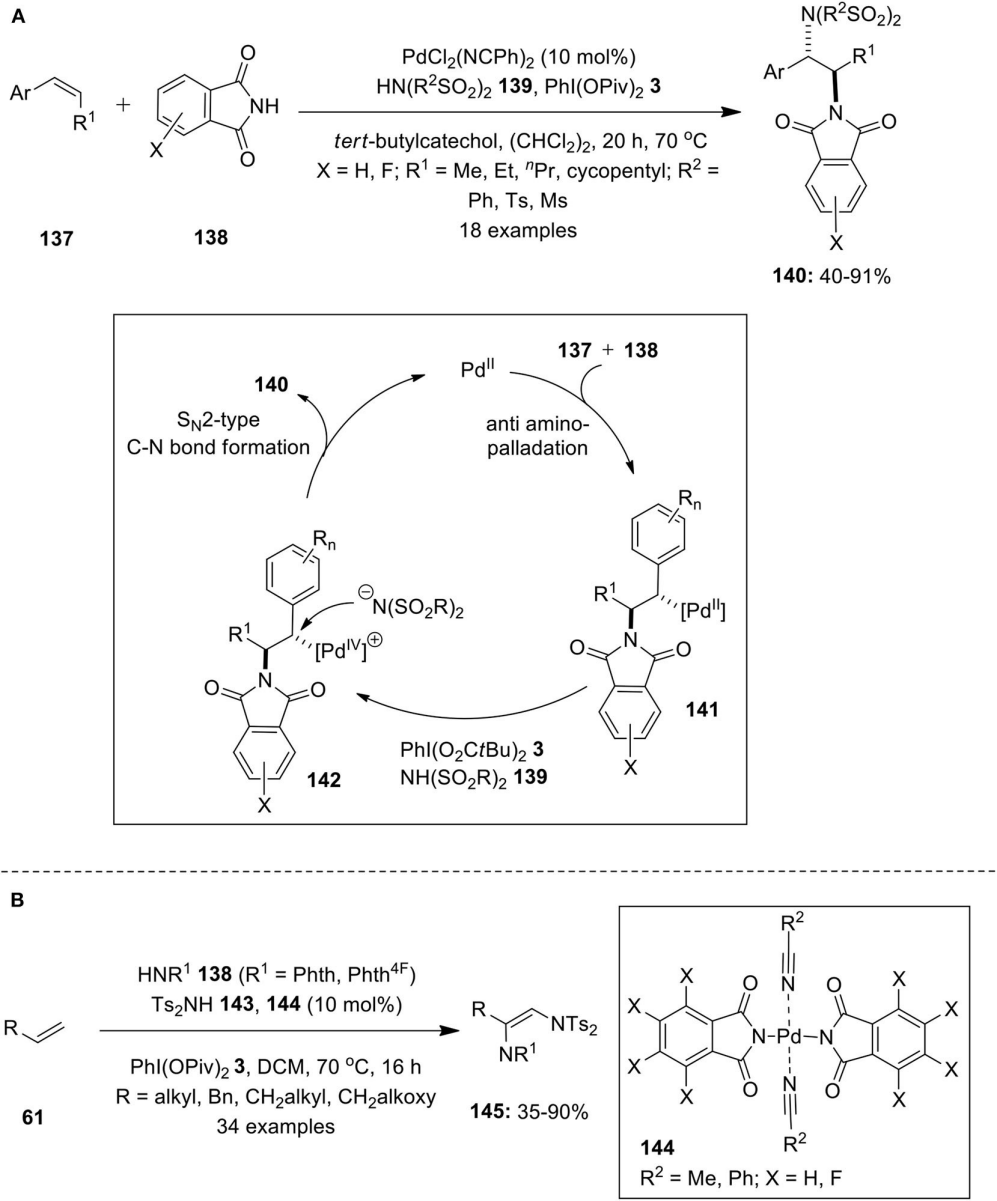
**(A)** Intermolecular diamination of internal alkenes **137** using Pd/PhI(OPiv)_2_-catalytic system and **(B)** Palladium-phthalimidato complex-catalyzed diamination of alkenes **61** using PhI(OPiv)_2_ **3** as oxidant.

Iglesias et al. developed the first example of Pd-catalyzed intermolecular diamination of terminal alkenes employing saccharin and bissulfonimides as the nitrogen sources in the presence of PhI(OPiv)_2_ **3** as an oxidant (Iglesias et al., 2010). Further allylic ethers were subjected to similar catalytic intermolecular 1,2-diamination reaction using nitrogen sources, phthalimide, and *N*-fluoro-bis(phenylsulfonyl)imide (Muniz et al., 2011). Later, Martinez and Muniz successfully employed Pd/PhI(OPiv)_2_-catalytic system for the intermolecular vicinal diamination of internal alkenes **137** with phthalimide **138** and bissulfonimides **139** as the two nitrogen sources (Martinez and Muniz, 2012). This method employs alkene as the limiting reagent and desired diamination products **140** were isolated in moderate to high yields with complete regio- and diastereoselectivity (Scheme 17A). The mechanistic approach involves initial alkene **137** co-ordination with the Pd catalyst followed by subsequent aminopalladation involving nucleophilic addition of **138** via *trans* stereochemistry to form δ-alkylpalladium complex **141**. Rapid oxidation of complex **141** gives Pd(IV) intermediate **142** which is attacked by bissulfonimide **139** to provide desired product **140** with net inversion of configuration at benzylic position.

In continuation, Martinez and Muniz reported the synthesis of new palladium-phthalimidato complexes **144** and demonstrated its broad applicability as catalysts in the vicinal diamination of alkenes **61** with phthalimide **143** and bissulfonimides **144** as nitrogen sources (Martinez and Muniz, 2016). The treatment of phthalimide **143** with Pd(OAc)_2_ in nitrile solution at room temperature resulted in the formation of palladium-phthalimidato complexes **144**. The air-stable preformed phthalimidato complexes proved to be versatile catalysts for the present diamination reaction providing desired products **145** in useful yields (Scheme 17B). Moreover, the same research group synthesized other bissaccharido palladium(II) complexes and investigated their application in catalytic regioselective diaminations and aminooxygenation of alkenes (Martinez et al., 2016).

**Scheme 18:**
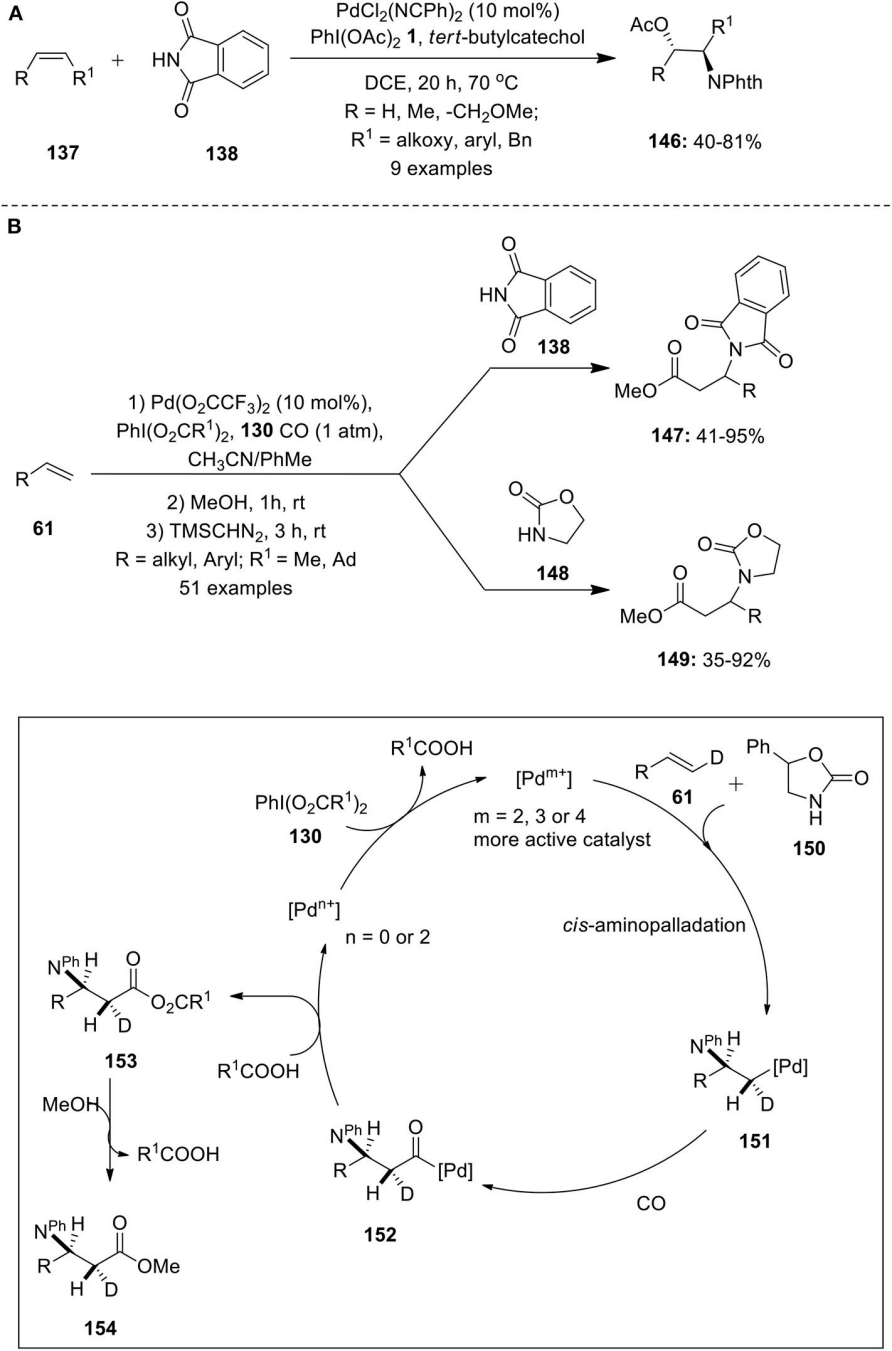
**(A)** Pd(II)-catalyzed PIDA-mediated aminoacetoxylation of alkenes **137** and **(B)** Pd(II)-catalyzed aminocarbonylation of alkenes **61** induced by iodine(III) reagent **130**.

Pd-catalyzed aminoacetoxylation of alkenes were independently reported by Sorensen and Stahl's groups employing 2 equivalence of olefin with respect to nitrogen nucleophiles (Alexanian et al., 2005; Liu and Stahl, 2006). Later, Muniz with his co-workers reported a modified method for an intermolecular aminoacetoxylation of internal/terminal alkenes **137** using alkene substrates as limiting reagent (Martinez et al., 2013). A series of alkenes such as allyl ethers, allyl benzenes, (Z)-β-methylstyrene, etc. were oxidized and converted into aminoacetoxylated product **146** using phthalimide **138** as a nitrogen source (Scheme 18A). Based on the experimental evidences, it was concluded that PhI(OAc)_2_ **1** influences the stereochemical aspect of the aminoacetoxylation reaction favoring *trans*-aminopalladation pathway.

In 2015, Cheng et al. have demonstrated an efficient and simple palladium-catalyzed protocol for the synthesis of β^−^ amino acid derivatives **147** and **149** from alkenes **61** via intermolecular aminocarbonylation reaction (Cheng J. et al., 2015). An array of aliphatic or aromatic terminal alkenes **61** were reacted with either phthalimide **138** or with 2-oxazolidone **148** under a carbon monoxide atmosphere in the presence of PhI(O_2_CR^1^)_2_ **130** as an oxidant (Scheme 18B). The reaction possessed excellent regioselectivity, broad substrates scope, and remarkable functional group tolerance. Reaction mechanism and stereochemistry was studied by taking deuterium labeled alkene **61** and substituted 2-oxazolidone **149** as an example. The reaction initiates with *cis*-aminopalladation of alkene **61** to form alkyl-Pd complex **151** and subsequent CO insertion gives intermediate **152**. Next, nucleophilic attack of carboxylate on to the acyl-Pd species **152** afforded anhydride product **153** which upon alcoholysis yields carboxylic ester **154**. Further the experimental evindence revealed that the iodine(III) reagent plays a crucial role in accelerating the intermolecular aminopalladation process.

**Scheme 19:**
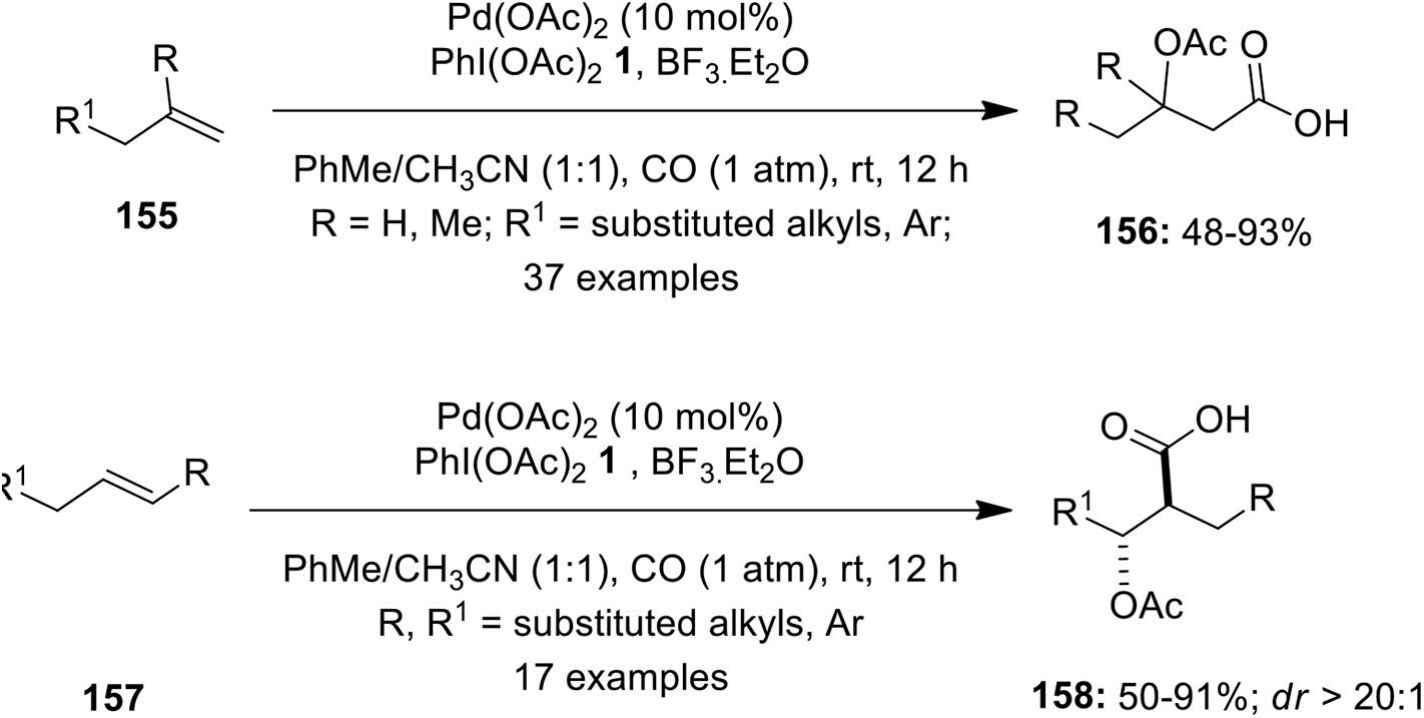
Pd(II)-catalyzed oxycarbonylation of alkenes **154** and **156** using PhI(OAc)_2_ **1** as an oxidant.

The same research group developed a novel Pd-catalyzed intermolecular oxycarbonylation of terminal **155** and internal alkenes **157** under CO atmosphere using PIDA **1** as oxidant (Li M. et al., 2016). This difunctionalization reaction leads to the facile synthesis of various β-oxycarboxylic acids **156** and **158** in useful yields with excellent functional group compatibility, regioselectivity, and diastereoselectivity (Scheme 19). The potential scope of this method was extended toward the synthesis of natural product, (+)-honaucin C in 48% yield with *ee* up to 99% (Li M. et al., 2016).

**Scheme 20:**
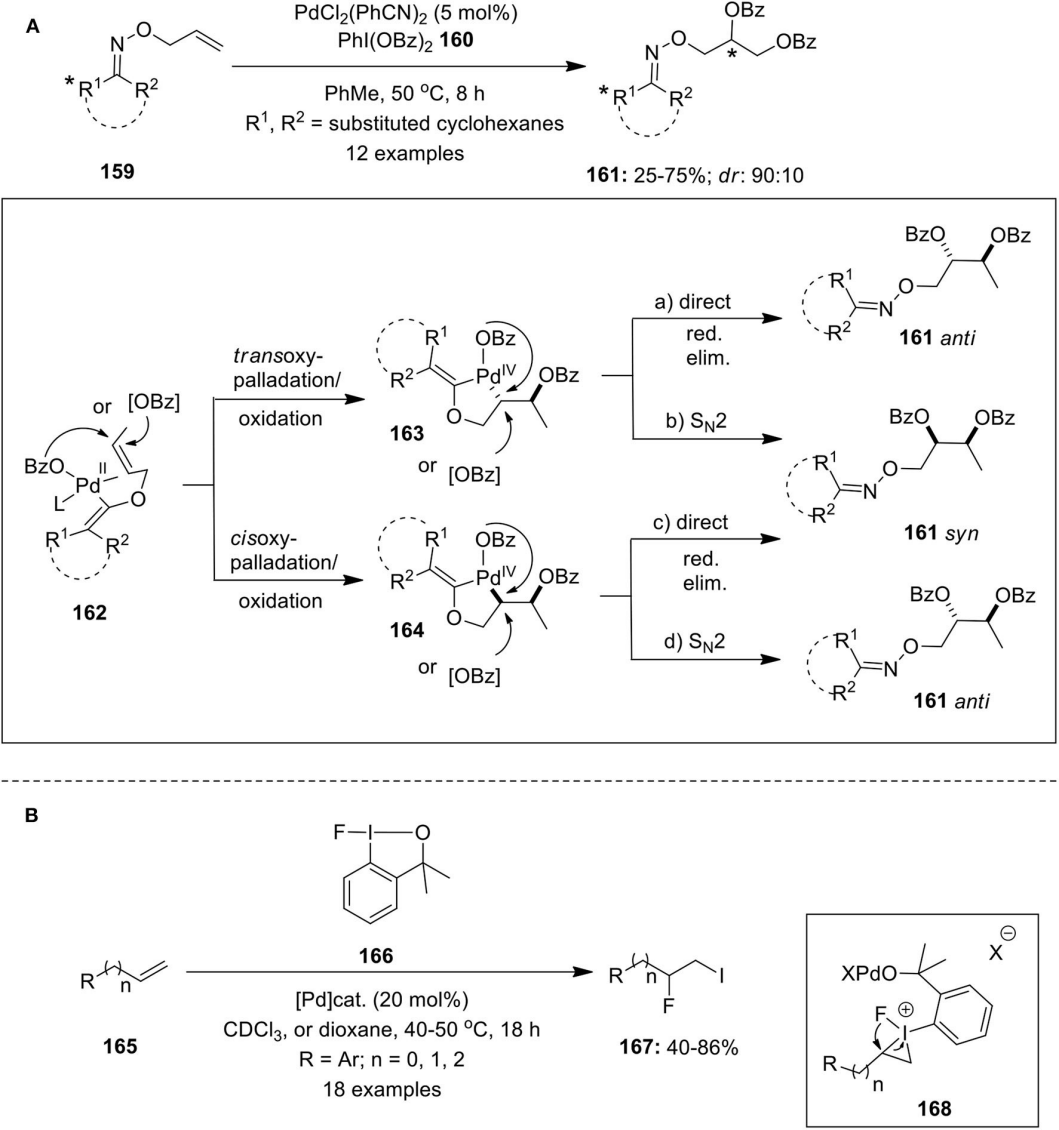
**(A)** Pd(II)-catalyzed asymmetric 1,2-dioxygenation chiral oxime allyl ether **107** using PhI(OBz)_2_ **160** and **(B)** Pd(II)-catalyzed iodofluorination of alkenes **110** employing fluoroiodane reagent **166**.

Vicinal alkene dioxygenation is an important method for the preparation of valuble 1,2-dioxygenated scaffolds. In this regard, Dong and Shi's research groups independently reported Pd-catalyzed vicinal dioxygenation of olefins employing hypervalent iodine reagent as terminal oxidant following distinct Pd^(II)^/Pd^(IV)^ mechanism (Li et al., 2008; Wang W. et al., 2010). Subsequently, Sanford's group developed Pd(II)-catalyzed chiral oxime-directed asymmetric 1,2-dioxygenation of alkenes **159** using PhI(OBz)_2_ **160** as an oxidant and benzoyloxy source (Neufeldt and Sanford, 2013). Various chiral allyl oxime ethers were screened and it was observed that menthone-derived substrates provided best reactivity and diastereoselectivity. This approach enabled efficient preparation of dibenzoylated products **161** with diasteriomeric ratio up to 9:1 (Scheme 20A). The proposed mechanism initiates with the coordination of oxime ether **159** with Pd catalyst to give intermediate **162** followed by subsequent oxypalladation and oxidation proceeding via either *trans* or *cis* geometry to deliver intermediate **163** or **164**. Final, reductive elimination could occur by direct (path “a” and “c”) or by S_N_2-type mechanisms (path b and d) that would lead to the formation of *syn* or *anti* products. Further, based on the experimental data authors suggested that reaction proceeds via *syn* addition and primary operative mechanism could be either “b” or “c”.

Furthermore, the Pd-catalyzed iodofluorination of alkenes **165** was reported by Ilchenko et al. employing fluoroiodane reagent **166** as iodine and fluorine sources (Ilchenko et al., 2016). The reaction was carried out using either Pd(BF_4_)_2_(MeCN)_4_ or PdCl_2_(MeCN)_2_ or Pd(OAc)_2_ as palladium catalysts in CDCl_3_ (Scheme 20B). The reaction proceeds via three-membered iodonium intermediate **168** which upon ring opening and subsequent C(sp^2^)–I bond cleavage delivers the desired iodofluorinated products **167**. Simple cycloalkenes such as cyclohexene and cycloheptene also undergoes iodofluorination reaction to give iodofluorinated products in modest yields.

## Conclusion

In conclusion, this review described various achievements made in palladium-catalyzed reactions mediated by hypervalent iodine compounds. Hypervalent iodine reagents are versatile, non-toxic, environment friendly, and easy to handle reagents in organic synthesis. In recent years, the use of hypervalent iodine reagents in palladium-catalyzed transformations has been widely studied as they are strong electrophiles and powerful oxidizing agents. Most of the reactions proceed via Pd(II/IV) redox cycles involving hypervalent iodine reagents-induced oxidation to generate Pd(IV) center as the key step. The inherent oxidizing nature and unique reactivity of these reagents with palladium catalysts enabled efficient preparation of synthetically useful molecules through C–O, C–N, C–C, C–Si, C–B, and C–halogen bond formation reactions. In addition, a number of Pd-catalyzed alkene difunctionalization reactions have been developed in recent times employing hypervalent iodine reagents. More importantly, oxidation of an unactivated C(sp^3^)–H bond has been successfully accomplished using a palladium and hypervalent iodine catalytic system which is a challenging problem. From future perspectives, palladium catalysis is well-established with respect to achiral synthesis, however reactions concerning to asymmetric synthesis are limited. Thus, development of enantioselective Pd-catalyzed reaction with hypervalent iodine reagents would be a much-anticipated area of research in the near future. Apart from this, use of recyclable polymer-supported hypervalent iodine reagents in palladium catalyzed reactions would be another interesting field of research.

## Author Contributions

All authors listed have made a substantial, direct and intellectual contribution to the work, and approved it for publication.

## Conflict of Interest

The authors declare that the research was conducted in the absence of any commercial or financial relationships that could be construed as a potential conflict of interest.
